# Chromosome Cohesion Established by Rec8-Cohesin in Fetal Oocytes Is Maintained without Detectable Turnover in Oocytes Arrested for Months in Mice

**DOI:** 10.1016/j.cub.2015.12.073

**Published:** 2016-03-07

**Authors:** Sabrina Burkhardt, Máté Borsos, Anna Szydlowska, Jonathan Godwin, Suzannah A. Williams, Paula E. Cohen, Takayuki Hirota, Mitinori Saitou, Kikuë Tachibana-Konwalski

**Affiliations:** 1 Institute of Molecular Biotechnology of the Austrian Academy of Sciences (IMBA), Vienna Biocenter Campus, Dr. Bohr-Gasse 3, Vienna 1030, Austria; 2 Department of Biochemistry, University of Oxford, South Parks Road, Oxford OX1 3QU, UK; 3 Nuffield Department of Obstetrics and Gynaecology, John Radcliffe Hospital, University of Oxford, Women’s Centre, Level 3, Oxford OX3 9DU, UK; 4 Department of Biomedical Sciences and Center for Reproductive Genomics, Cornell University, Tower Road, Ithaca, NY 14853, USA; 5 Department of Anatomy and Cell Biology, Graduate School of Medicine, Kyoto University, Yoshida-Konoe-cho, Sakyo-ku, Kyoto 606-8501, Japan; 6 JST, ERATO, Yoshida-Konoe-cho, Sakyo-ku, Kyoto 606-8501, Japan

**Keywords:** cohesin, meiosis, oocytes

## Abstract

Sister chromatid cohesion mediated by the cohesin complex is essential for chromosome segregation in mitosis and meiosis [1]. Rec8-containing cohesin, bound to Smc3/Smc1α or Smc3/Smc1β, maintains bivalent cohesion in mammalian meiosis [2, 3, 4, 5, 6]. In females, meiotic DNA replication and recombination occur in fetal oocytes. After birth, oocytes arrest at the prolonged dictyate stage until recruited to grow into mature oocytes that divide at ovulation. How cohesion is maintained in arrested oocytes remains a pivotal question relevant to maternal age-related aneuploidy. Hypothetically, cohesin turnover regenerates cohesion in oocytes. Evidence for post-replicative cohesion establishment mechanism exists, in yeast and invertebrates [7, 8]. In mouse fetal oocytes, cohesin loading factor Nipbl/Scc2 localizes to chromosome axes during recombination [9, 10]. Alternatively, cohesion is maintained without turnover. Consistent with this, cohesion maintenance does not require Smc1β transcription, but unlike Rec8, Smc1β is not required for establishing bivalent cohesion [11, 12]. Rec8 maintains cohesion without turnover during weeks of oocyte growth [3]. Whether the same applies to months or decades of arrest is unknown. Here, we test whether Rec8 activated in arrested mouse oocytes builds cohesion revealed by TEV cleavage and live-cell imaging. Rec8 establishes cohesion when activated during DNA replication in fetal oocytes using tamoxifen-inducible Cre. In contrast, no new cohesion is detected when Rec8 is activated in arrested oocytes by tamoxifen despite cohesin synthesis. We conclude that cohesion established in fetal oocytes is maintained for months without detectable turnover in dictyate-arrested oocytes. This implies that women’s fertility depends on the longevity of cohesin proteins that established cohesion in utero.

## Highlights

• Rec8-mediated chromosome cohesion is established in fetal oocytes
• Bivalent cohesion is maintained without detectable turnover in arrested oocytes
• Irreversible cohesin loss underlies age-related chromosome missegregation

## Graphical Abstract

**Figure:**
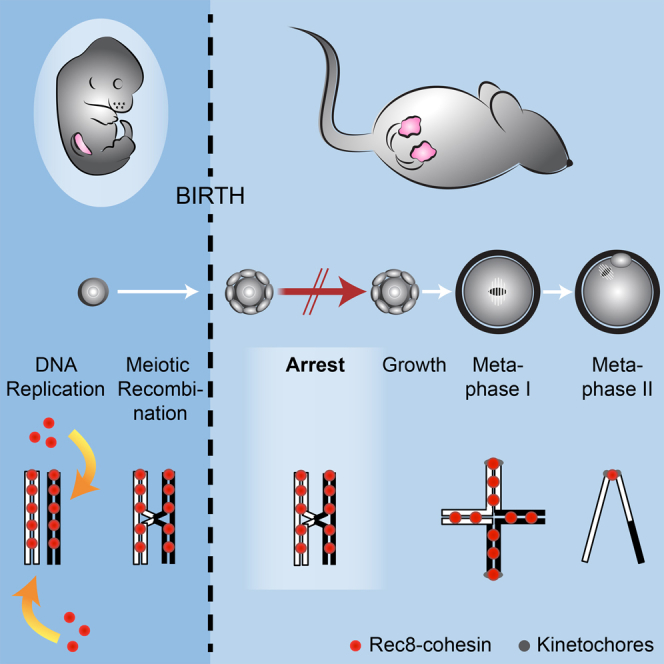

## Teaser

How chromosome cohesion is maintained in female germ cells arrested for months or decades is poorly understood. Burkhardt et al. show that cohesion is built in fetal oocytes and after birth is maintained without detectable renewal for months. This implies that the oocyte’s inability to renew cohesion contributes to maternal age-related trisomies.

## Results and Discussion

**Figure 1:**
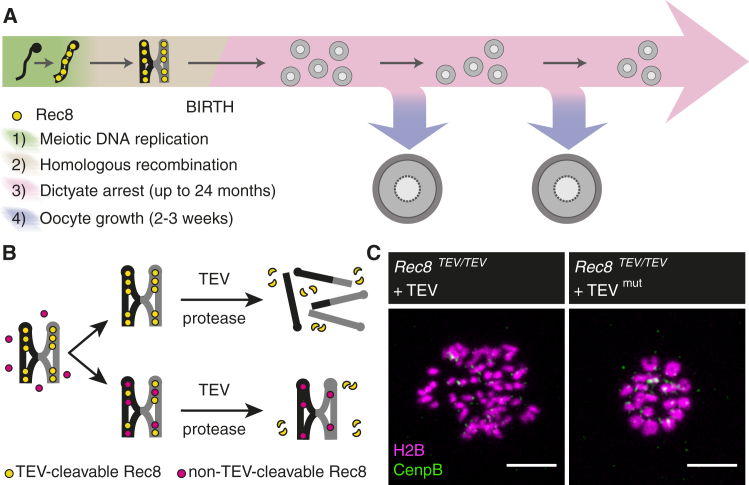
Probing Cohesion Maintenance during Mammalian Female Meiosis Using a Functional Cohesion Assay (A) Mammalian female meiosis can be structured into four stages: (1) meiotic DNA replication in which sister chromatid cohesion is presumably established, (2) meiotic recombination in which reciprocal recombination of homologous chromosomes (black and gray) results in crossovers that manifest as chiasmata, (3) the prolonged resting state at the dictyate stage of prophase I after birth, and (4) the growing phase that starts when an oocyte is recruited from the resting pool and leads to formation of a mature oocyte. The duration of the dictyate arrest and growing phase correspond to mouse. Schematic is not drawn to scale. (B) Schematic of cohesion rescue assay. TEV-cleavable Rec8 (yellow) establishes and maintains bivalent cohesion. Non-TEV-cleavable Rec8 (red) is activated after meiotic cohesion establishment. Cleavage by TEV protease reveals whether de novo expressed non-TEV-cleavable Rec8 is able to build functional cohesive structures: cleavage of bivalents into single chromatids indicates no functional loading, while resistance to TEV cleavage reveals functional cohesive structures entrapping sister DNA molecules. (C) *Rec8*^*TEV/TEV*^ oocytes are microinjected with mRNA encoding H2B-mCherry, CenpB-EGFP, and TEV protease. Confocal time-lapse microscopy allows scoring of chromosome type at metaphase I (5 hr post-GVBD). TEV protease efficiently converts bivalents to chromatids, which are detected as at least 72 single chromatids and no bivalents (oocytes analyzed n = 40), while no cleavage of all 20 bivalents is observed using mutant TEV protease (TEV^mut^; oocytes analyzed n = 16). Scale bar, 10 μm. See also Figure S1.

The frequency of clinically recognized trisomic pregnancies increases with maternal age [13]. Most aneuploid pregnancies arise as a consequence of chromosome segregation errors during the first meiotic division of female germ cells called oocytes, leading to aneuploid eggs [13, 14, 15]. On average, 20% of human eggs and 1%–2% of mouse eggs are aneuploid [14]. In aging human and mouse oocytes, cohesin levels decrease, centromeric cohesion weakens, and chromosome segregation errors increase [16, 17, 18, 19, 20, 21]. To gain insights into age-related chromosome missegregation, we need a molecular understanding of cohesion establishment and maintenance in oocytes. A defining feature of mammalian oocytes is the prolonged arrest at the dictyate stage of prophase I that lasts for months in the mouse and decades in the human (Figure 1A). Crucially, it is not known whether bivalent cohesion is maintained with or without turnover during the arrest. If cohesion is maintained by cohesin turnover, then either the cohesion establishment mechanism deteriorates or the cohesin pool needed for replenishment diminishes in aging oocytes (Figure S1A). Alternatively, if cohesion is maintained without cohesin turnover, then cohesin loss from chromosomes is irreversible (Figure S1B). Either model is interesting and has the potential to explain what goes awry in aging oocytes, leading to the production of aneuploid fetuses.

### A Functional Cohesion Rescue Assay in Meiosis I Oocytes

The entrapment of sister DNA molecules by cohesin complexes can be measured indirectly and directly using biochemical and cell biological approaches [7, 22, 23, 24]. To determine whether cohesion is maintained with or without building additional cohesive structures after DNA replication, we used a functional cohesion assay that we had established previously (Figure 1B) [3]. Briefly, endogenous Rec8 contains engineered Tobacco Etch Virus (TEV) recognition sites rendering cohesin cleavable by TEV protease. TEV protease expression in *Rec8*^*TEV/TEV*^ oocytes converts 100% of bivalents to chromatids. To test whether new cohesion is built, Rec8-Myc that is not cleavable by TEV protease is induced in addition to endogenous Rec8TEV that establishes cohesion during DNA replication (Figure 1B). If Rec8-Myc becomes incorporated into cohesin complexes that establish cohesion, then bivalents become resistant to destruction by TEV protease. If no new cohesion is established, then TEV cleavage of Rec8 converts bivalents to chromatids. We assume that over time slow cohesin decay takes place and contemporaneously occurring reloading of Rec8-Myc can be revealed by TEV cleavage to rapidly destroy endogenous Rec8. The genetic components are *Rec8*^*TEV/TEV*^ oocytes that also contain a conditional silent *BAC* transgene with a *Stop* cassette flanked by *LoxP* sites, *(Tg)Stop/Rec8-Myc*. Cre recombinase deletes the *Stop* cassette and activates *Rec8-Myc* transgene expression. Deletion of the *Stop* cassette using *(Tg)Sox2-*Cre in the early embryo, before primordial germ cell specification, rescues bivalent cohesion in mature oocytes [3, 25]. Therefore, Rec8-Myc is capable of establishing functional cohesion when activated before meiosis.

To visualize the cohesion status of chromosomes in meiosis I oocytes, we used a microinjection and live-cell imaging approach. Mature germinal vesicle (GV) stage oocytes isolated from sexually mature females are cultured in 3-isobutyl-1-methylxanthine (IBMX)-containing medium to inhibit germinal vesicle breakdown (GVBD). GV oocytes are microinjected with mRNA encoding H2B-mCherry to visualize chromosomes, TEV protease and another marker such as CenpB-EGFP that localizes to kinetochores. After expression of the mRNA constructs, oocytes are released into IBMX-free medium to resume meiosis and followed by confocal time-lapse microscopy. Bivalents are converted to chromatids within 3–4 hr in *Rec8*^*TEV/TEV*^ oocytes expressing wild-type but not mutant TEV protease (TEV^mut^), demonstrating that bivalent cohesion is intact without TEV cleavage (Figure 1C). Therefore, the comparison of bivalents versus chromatids enables the visualization of functional cohesion in live oocytes.

### *Gdf9*-iCre and *Spo11*-Cre Delete during Meiotic DNA Replication

**Figure 2:**
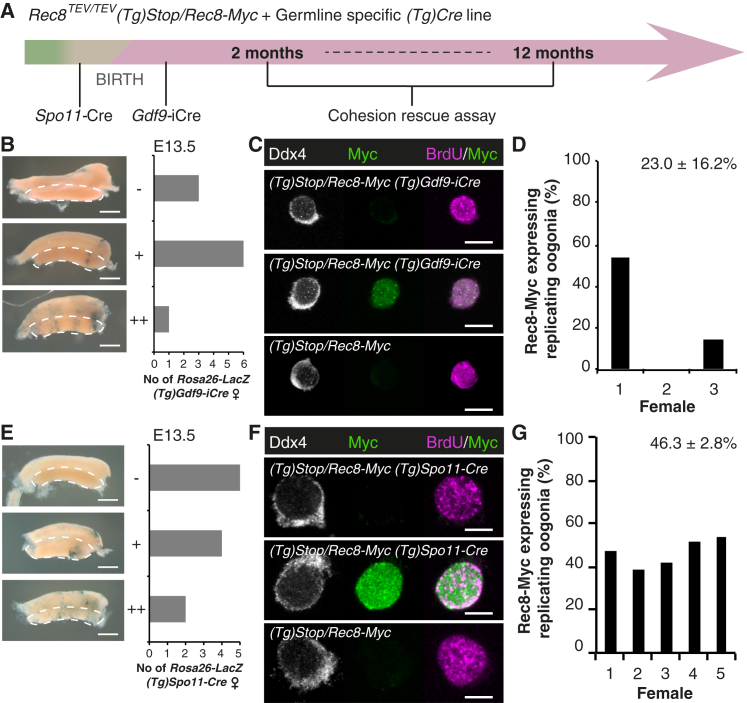
*Gdf9*-iCre and *Spo11*-Cre Activate Rec8-Myc during Meiotic DNA Replication (A) Interpretation of the cohesion rescue assay requires activation of the *Rec8-Myc* transgene after meiotic DNA replication (green). Thus, *Gdf9*-iCre or *Spo11*-Cre was used to activate Rec8-Myc in oocytes shortly after birth (dictyate stage, pink) or during homologous recombination (beige), respectively. (B) Timely deletion analysis of *Gdf9*-iCre. Scoring of X-gal positive cells in *Rosa26-LacZ (Tg)Gdf9-iCre* embryonic ovaries at E13.5 according to classification into negative (−), weakly positive (+), and positive (++); n = 10 female embryos. The dashed line indicates the gonad. Scale bar, 1 mm. (C and D) *Gdf9*-iCre activates Rec8-Myc during meiotic S phase. Embryonic day E13.5 *(Tg)Stop/Rec8-Myc (Tg)Gdf9-iCre* embryonic ovaries were scored for Rec8-Myc expressing replicating (BrdU-positive) oogonia identified by germ cell-specific cytoplasmic Ddx4 staining; n = 3 females. No Myc signal was observed in oogonia from control *(Tg)Stop/Rec8-Myc* females; n = 2 females. (C) Representative images; scale bar, 10 μm. (D) Quantification for *(Tg)Stop/Rec8-Myc (Tg)Gdf9-iCre* embryonic ovaries; n > 100 cells per female. Mean ± SEM given. (E–G) Timely deletion analysis of *Spo11*-Cre. (E) Scoring of X-gal positive cells in *Rosa26-LacZ (Tg)Spo11-Cre* embryonic ovaries at E13.5 according classification into negative (–), weakly positive (+), and positive (++); n = 11 female embryos. The dashed line indicates the gonad. Scale bar, 1 mm. (F and G) *Spo11*-Cre activates Rec8-Myc during meiotic S phase. Embryonic day E14.5 *(Tg)Stop/Rec8-Myc (Tg)Spo11-Cre* embryonic ovaries were scored for Rec8-Myc expressing replicating (BrdU-positive) oogonia identified by germ cell-specific cytoplasmic Ddx4 staining; n = 5 females. No Myc signal was observed in oogonia from control *(Tg)Stop/Rec8-Myc* females; n = 3 females. (F) Representative images; scale bar, 10 μm. (G) Quantification for *(Tg)Stop/Rec8-Myc (Tg)Spo11-Cre* embryonic ovaries; n > 500 cells per female. Mean ± SEM given. See also Figure S2 and Table S1.

To test whether cohesion is maintained with or without turnover during the dictyate arrest, we sought to activate the *Rec8-Myc* transgene in arrested oocytes shortly after birth. To achieve this, we selected the widely used deleter strain *(Tg)Gdf9-iCre* in which Cre expression is controlled by the promoter of growth differentiation factor 9 (Gdf9) [11, 26, 27, 28, 29, 30, 31, 32, 33, 34]. *(Tg)Gdf9-iCre* is thought to delete conditional alleles in oocytes shortly after birth, i.e., days after DNA replication and meiotic recombination (Figure 2A).

Since it is important that *Rec8-Myc* transgene activation occurs after meiotic DNA replication, we carried out due diligence to confirm that *Gdf9*-iCre does not delete during DNA replication. Using a conditional LacZ reporter strain (*Rosa26-LacZ*) [35], we analyzed *Rosa26-LacZ (Tg)Gdf9-iCre* ovaries on embryonic day E13.5 when oocytes enter meiosis. Unexpectedly, seven out of ten fetal ovaries contained X-gal positive cells (Figure 2B), suggesting that deletion might occur as early as meiotic DNA replication. Indeed, Rec8-Myc is expressed in up to 50% of replicating germ cells, identified as BrdU- and Ddx4-positive cells, in oocytes from *(Tg)Stop/Rec8-Myc (Tg)Gdf9-iCre* females (Figures 2C and 2D). *Gdf9*-iCre activated Rec8-Myc before or during meiotic DNA replication in two out of three female embryos (Figure 2D). Overall *Gdf9*-iCre deletes with high efficiency (Table S1), but the deletion timing varies between mice and between oocytes within one mouse. In agreement with this, cohesion rescue experiments using oocytes from *Rec8*^*TEV/TEV*^ *(Tg)Stop/Rec8-Myc (Tg)Gdf9-iCre* females resulted in variable rescue efficiencies (Figures S2A and S2B). It is not possible to know whether cohesion rescue in these cells is due to cohesion establishment during DNA replication or thereafter. On a technical note, while it cannot be excluded that mouse strain background and genetic locus might have some effect on the timing of Cre-mediated deletion, our analyses using two different target loci showing earlier deletion than previously thought raise concerns about the suitability of *Gdf9*-iCre for cell cycle phase-specific deletion studies.

A second approach to activate the *Rec8-Myc* transgene after meiotic DNA replication might rely on Cre recombinase controlled by a promoter driving expression of a protein required for recombination. The topoisomerase-like enzyme Spo11 generates DNA double-strand breaks that initiate recombination [36]. Therefore, we chose to test *Spo11*-Cre. If *Spo11*-Cre deletes after DNA replication and before or during recombination, then this system will test whether cohesion is built at all after DNA replication. It would not be possible to distinguish whether cohesion is generated during recombination or the dictyate-stage arrest.

To characterize the timing of *Spo11*-Cre, we used *Rosa26-LacZ* and *(Tg)Stop/Rec8-Myc* mice (Figures 2E–2G). *Spo11*-Cre also deletes during meiotic DNA replication in 46% of replicating oocytes (Figure 2G). In agreement with this, cohesion rescue is detected in some oocytes from *Rec8*^*TEV/TEV*^ *(Tg)Stop/Rec8-Myc Spo11-*Cre females (Figures S2C and S2D). We conclude that neither *Gdf9*-iCre nor *Spo11*-Cre allow consistent activation of Rec8-Myc after meiotic DNA replication in fetal oocytes.

### Timely Controlled Rec8 Activation in Fetal and Adult Arrested Oocytes

To demonstrate whether arrested oocytes maintain cohesion with or without turnover, it is important to activate *Rec8-Myc* transgene expression after meiotic DNA replication and homologous recombination. Since there are currently no mouse strains other than *(Tg)Gdf9*-iCre that are thought to delete in arrested oocytes before growth, we chose to directly control the timing of Cre-mediated deletion by injection of 4-hydroxytamoxifen (4-OHT). Specifically, the germ cell-specific *Dppa3* promoter drives expression of Cre fused to mouse estrogen receptors (MERCreMER) and a PEST degradation motif in *(Tg)Dppa3-MCM-P* mice [37] (Table S1). MERCreMER is cytoplasmic, and 4-OHT binding to the receptors triggers translocation of the Cre-fusion to the nucleus, facilitating timely genetic deletion [38].

The challenges with this approach are a risk of deletion without 4-OHT, inefficient deletion with 4-OHT, and effects of 4-OHT on fertility. Since background deletion could result in a false positive cohesion rescue, we first tested whether there is any deletion without 4-OHT. Reassuringly, vehicle injection into *Rosa26-LacZ (Tg)Dppa3-MCM-P* females resulted in no X-gal positive oocytes in ovary sections, consistent with the negligible background reported by others (Figure S3). On the other hand, 4-OHT injection into *Rosa26-LacZ (Tg)Dppa3-MCM-P* females resulted in ∼25% X-gal positive oocytes, indicating that Cre-mediated deletion had occurred (Figure S3). Since the deletion efficiency is low, oocytes with a deleted *Stop* cassette in *Rec8-Myc* will be identified by single-cell PCR genotyping after the cohesion rescue assay (Table S1). Lastly, 4-OHT-injected *(Tg)Stop/Rec8-Myc (Tg)Dppa3-MCM-P* females are fertile for up to 12 litters, and 28% of offspring inherited *(Tg)Rec8-Myc* (n = 3 females + 4-OHT; n = 3 females + vehicle). Therefore, 4-OHT and Cre-mediated deletion do not grossly affect oocyte maturation and female fertility.

**Figure 3:**
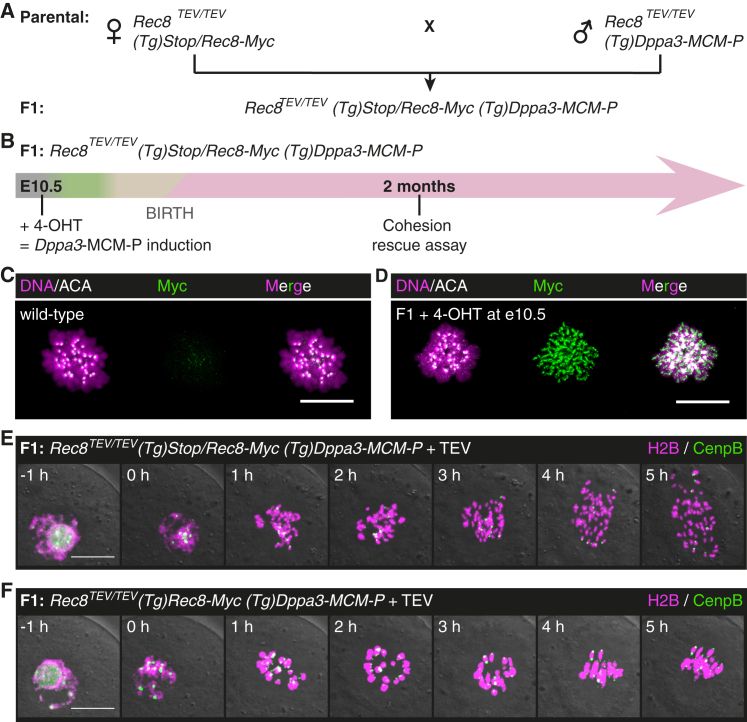
Rec8 Activated in Fetal Oocytes Builds Cohesive Structures (A) Mating scheme to obtain female F1 offspring of the required genotype *Rec8*^*TEV/TEV*^*(Tg)Stop/Rec8-Myc (Tg)Dppa3-MCM-P*. (B) Activation of Rec8-Myc during meiotic S phase in fetal oocytes. Pregnant *Rec8*^*TEV/TEV*^*(Tg)Stop/Rec8-Myc* females are injected with 4-OHT on embryonic day E10.5 to induce deletion by *Dppa3*-MCM-P in embryos. Oocytes are isolated from F1 females with the appropriate genotype. Green, meiotic DNA replication; beige, homologous recombination; pink, dictyate stage. (C and D) Metaphase I chromosome spread showing localization of Rec8-Myc to bivalent chromosomes in oocytes from (C) wild-type and (D) F1 *Rec8*^*TEV/TEV*^*(Tg)Stop/Rec8-Myc (Tg)Dppa3-MCM-P* female; oocytes analyzed n = 9 and n = 7, respectively. Centromeres are marked by anti-centromere antibody (ACA). Scale bar, 20 μm. (E and F) Representative still images of oocytes isolated from F1 *Rec8*^*TEV/TEV*^*(Tg)Stop/Rec8-Myc (Tg)Dppa3-MCM-P* females microinjected with mRNA encoding H2B-mCherry, CenpB-EGFP, and TEV protease. (E) Bivalents are converted to chromatids in cells without *Stop* cassette deletion; n = 17 oocytes. (F) Bivalents are retained in oocytes with successful deletion of the *Stop* cassette; n = 5 oocytes. Genotype of single cells was confirmed after live-cell imaging. Oocytes were obtained from >3 females. Scale bar, 20 μm. See also Figure S3 and Table S1.

To test whether 4-OHT induces sufficient levels of Rec8 to rescue bivalent cohesion in meiosis I oocytes, we embarked on a novel fetal-to-adult oocyte experiment. The idea is to activate Rec8 in fetal oocytes and perform the cohesion rescue assay on the same oocytes isolated from the adult female. Pregnant *Rec8*^*TEV/TEV*^ *(Tg)Stop/Rec8-Myc* females mated to *Rec8*^*TEV/TEV*^ *(Tg)Dppa3-MCM-P* males were injected with 4-OHT on embryonic day E10.5 to activate Rec8-Myc in replicating oocytes of female embryos (F1 generation) (Figure 3A). *Rec8*^*TEV/TEV*^ *(Tg)Stop/Rec8-Myc (Tg)Dppa3-MCM-P* F1 females were sacrificed at 2 months for oocyte isolation (Figure 3B). If Rec8-Myc activated by 4-OHT established cohesion in a replicating fetal oocyte, then the same oocyte isolated from the adult F1 female would be expected to express Rec8-Myc. Indeed, Rec8-Myc localized to bivalents on a meiosis I chromosome spread from an oocyte isolated from a *Rec8*^*TEV/TEV*^ *(Tg)Stop/Rec8-Myc (Tg)Dppa3-MCM-P* F1 female (Figures 3C and 3D). The localization of cohesin to the inter-chromatid axis of bivalents suggests, but does not demonstrate, that cohesin is entrapping sister chromatids. To test for functional cohesion, we injected oocytes with TEV protease and imaged them. Indeed, bivalent cohesion is rescued in oocytes from *Rec8*^*TEV/TEV*^ *(Tg)Stop/Rec8-Myc (Tg)Dppa3-MCM-P* F1 females with a deleted *Stop* cassette (Figures 3E and 3F). This implies that sufficient levels of Rec8 are synthesized due to 4-OHT to establish cohesion and rescue bivalent cohesion in adult oocytes, at least when Rec8 is activated in fetal oocytes.

**Figure 4:**
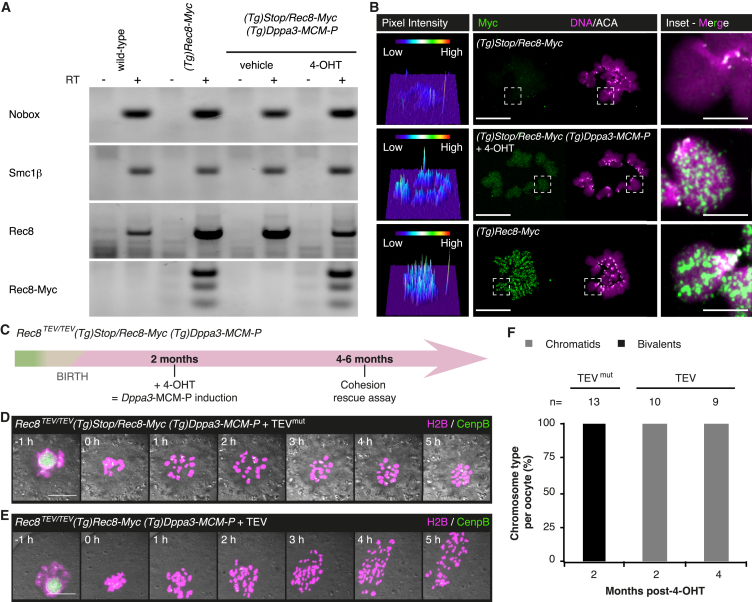
Bivalent Cohesion Is Maintained without Detectable Turnover of Rec8-Cohesive Structures for Months during the Dictyate Arrest (A) Rec8-Myc is transcribed in adult ovary. Detection of mRNA for Nobox, Smc1β, Rec8, and Rec8-Myc by RT-PCR from adult ovary. RT, reverse transcriptase. See also Figure S4. (B) Rec8-Myc is synthesized in oocytes from adult *Rec8*^*TEV/TEV*^*(Tg)Stop/Rec8-Myc (Tg)Dppa3-MCM-P* female analyzed 2 months post-4-OHT. Metaphase I chromosome spread showing expression and localization of Rec8-Myc to bivalent chromosomes. Left panel shows 3D surface plots of Rec8-Myc pixel fluorescence intensities. Centromeres are marked by ACA. Oocytes analyzed from top to bottom: n = 11, 7, 16. Inset has been brightness and contrast enhanced equally in all three panels. Scale bar, 20 μm; inset scale bar, 5 μm. (C) Timing of cohesion rescue assay utilizing *Dppa3*-MCM-P to activate Rec8-Myc in adult female mice. Green, meiotic DNA replication; beige, homologous recombination; pink, dictyate stage. (D and E) Representative still images of oocytes isolated from *Rec8*^*TEV/TEV*^*(Tg)Stop/Rec8-Myc (Tg)Dppa3-MCM-P* females microinjected with mRNA encoding H2B-mCherry, CenpB-EGFP, and TEV protease. (D) Bivalents are retained in oocytes microinjected with mutant TEV mRNA. (E) TEV protease converts bivalents to chromatids in cells with successful *Stop* cassette deletion. Single-cell genotyping was performed after live-cell imaging. Scale bar, 20 μm. (F) Quantification of cohesion rescue assay in oocytes from *Rec8*^*TEV/TEV*^*(Tg)Stop/Rec8-Myc (Tg)Dppa3-MCM-P* females with successful *Stop* cassette deletion used 2 or 4 months post-4-OHT treatment. Oocytes were obtained from >3 females; n = number of oocytes analyzed. See also Figure S3; Table S1; Movies S1 and S2.

Before using the timely controlled activation of Rec8 in dictyate-arrested oocytes, it was necessary to test whether Rec8 is transcribed in arrested oocytes. Rec8 transcripts are detectable in adult ovaries [39] (Figures 4A and S4), but these could be stored mRNA that was synthesized in early meiosis. To test whether Rec8-Myc is de novo transcribed in post-recombination oocytes, we injected *(Tg)Stop/Rec8-Myc (Tg)Dppa3-MCM-P* adult females with vehicle or 4-OHT and performed RT-PCR on whole ovaries. *(Tg)Rec8-Myc* ovaries served as a positive control to identify Rec8-Myc mRNA; the presence of oocytes was confirmed using the germ cell-specific transcripts Nobox and Smc1β. Rec8-Myc was expressed specifically in 4-OHT- and not in vehicle-injected *(Tg)Stop/Rec8-Myc (Tg)Dppa3-MCM-P* ovaries (Figures 4A and S4). Since *Rec8-Myc* is under control of the endogenous promoter in the *BAC*, de novo transcription of Rec8-Myc suggests that endogenous Rec8 is also transcribed in adult oocytes.

We next tested whether Rec8 protein is synthesized in adult oocytes. While no Rec8-Myc signal was detectable on chromosome spreads from control *(Tg)Stop/Rec8-Myc* oocytes, little but detectable Rec8-Myc localized to chromosomes from oocytes isolated from *(Tg)Stop/Rec8-Myc (Tg)Dppa3-MCM-P* females injected with 4-OHT (Figure 4B). We conclude that Rec8 protein is synthesized de novo and associates with chromosomes in adult oocytes.

In the key experiment, we asked whether cohesion is maintained with or without turnover in oocytes arrested for months at the dictyate stage of prophase I. *Rec8*^*TEV/TEV*^ *(Tg)Stop/Rec8-Myc (Tg)Dppa3-MCM-P* adult females were injected with 4-OHT to activate Rec8-Myc in oocytes, and the cohesion rescue assay was performed 2 or 4 months after activation (Figure 4C). Following TEV protease injection and time-lapse imaging, single-cell PCR identified 46% of oocytes with activated Rec8-Myc (n = 71 oocytes, 8 females, Table S1). Importantly, 100% of oocytes with activated Rec8-Myc both after 2 or 4 months displayed chromatids in meiosis I (Figures 4D–4F; Movies S1 and S2). Therefore, cohesion is maintained without turnover using newly synthesized Rec8 in oocytes arrested for several months at the dictyate stage of prophase I. Current experiments do not allow us to exclude that Rec8-cohesin complexes assembled early in meiosis might turn over. In summary, we conclude that cohesive structures maintaining bivalent cohesion must have been built before *(Tg)Dppa3*-MCM-P activation, most likely during DNA replication. Since genetic tools like *Spo11*-Cre cannot distinguish temporally between meiotic DNA replication and homologous recombination (Figures 2F, 2G, and S2E), it remains an open question whether additional cohesive structures are built during meiotic recombination or whether cohesion is established exclusively during DNA replication.

### Conclusions

Overall our results show that sister chromatid cohesion mediated by Rec8-containing cohesin complexes is established in fetal oocytes and maintained without detectable turnover after birth, both during the prolonged dictyate-stage arrest and the weeks of oocyte growth [3]. To fully understand cohesin dynamics in female meiosis will require an integrated approach including other cohesin complexes and a variety of techniques. We have employed a functional cohesion rescue assay that overcomes some limitations of cohesin detection by indirect immunofluorescent staining of chromosomes, which could reflect any mode of association, e.g., binding to chromatids (non-cohesive) or holding sister chromatids together (cohesive). Our results are not mutually exclusive with the findings that the cohesin loading factor Nipbl/Scc2 localizes to chromosome axes during meiotic recombination since we have investigated post-recombination, dictyate-stage arrested oocytes [9, 10]. Moreover, it is conceivable that Nipbl/Scc2 loads different types of cohesin complexes, which may contain Rad21L rather than Rec8, onto chromosomes. Thus, it remains an open question whether cohesion is built during meiotic recombination.

The advantage of the TEV cleavage assay of cohesin combined with an inducible transgenic rescue construct is that cohesion of sister chromatids is revealed in live cells. At the same time, it is challenging to empirically determine the sensitivity of the assay toward newly built cohesion. Certainly 50% turnover is robustly detected as bivalents remain intact following TEV protease expression in *Rec8*^*TEV/+*^ oocytes [3]. Given that as little as 13% of cohesin is sufficient for cohesion in yeast [40], it is likely that relatively few cohesin molecules mediating cohesion would be sufficient for rescue of bivalent chromosomes. It is therefore noteworthy that Rec8-Myc expression is controlled by the endogenous promoter on the *BAC*, and we have investigated the mechanism of cohesion maintenance relevant to the wild-type.

The discovery that bivalent cohesion is established predominantly, if not exclusively, in fetal oocytes has important implications for aging oocytes with increasing chromosome segregation errors [17, 18, 19, 20, 21]. Rather than invoking deterioration of cohesion establishment mechanisms or diminishing soluble cohesin proteins, our results suggest that age-related chromosome missegregation is due to the irreplaceable loss of cohesin complexes holding chromosomes together (Figure S1B). How cohesion is maintained at a mechanistic level for a long time and whether new cohesion is actively prevented by anti-establishment factors such as Wapl remain key questions for the future. Our work in mouse female meiosis supports the hypothesis that the inability of oocytes to build cohesion during the dictyate arrest that lasts for months or decades contributes to maternal age-related chromosome missegregation and production of aneuploid fetuses.

For material and methods, see Supplemental Information. The usage of mice followed the international guiding principles for biomedical research involving animals (the Council for International Organizations of Medical Sciences) and were in agreement with the authorizing committee.

## Author Contributions

Conceptualization, K.T.-K., S.B., and M.B.; Methodology, K.T.-K., S.B., M.B., and S.A.W.; Formal Analysis, S.B. and M.B.; Investigation, K.T.-K., S.B., M.B., and A.S.; Resources, J.G., P.E.C., M.S., and T.H.; Writing – Original Draft, K.T.-K.; Writing – Review & Editing, K.T.-K., S.B., and M.B.; Visualization, S.B.; Supervision, K.T.-K.; Project Administration, K.T.-K.; Funding Acquisition, K.T.-K.

## Supplemental Information

Document S1. Supplemental Experimental Procedures, Figures S1–S4, and Table S1

Movie S1. Oocyte with Activated *Rec8-Myc* Transgene Displays Conversion of Bivalents to Chromatids by TEV Cleavage, Related to Figure 4 Live-cell imaging of one oocyte isolated from a *Rec8*^*TEV/TEV*^*(Tg)Stop/Rec8-Myc (Tg)Dppa3-MCM-P* female (2 months post-4-OHT treatment) expressing H2B-mCherry (magenta) and TEV protease. Deletion of the *Stop* cassette was confirmed by single-cell genotyping post-imaging. Time intervals 0.5 hr. The scale bar represents 20 μm.

Movie S2. Bivalents Are Retained in Oocytes When Using Mutant TEV Protease, Related to Figure 4 Live-cell imaging of one *Rec8*^*TEV/TEV*^ oocyte expressing H2B-mCherry (magenta) and mutant TEV protease. Time intervals 0.5 hr. The scale bar represents 20 μm.

Document S2. Article plus Supplemental Information
