# Supplementary material for: Chromosome Cohesion Established by Rec8-Cohesin in Fetal Oocytes Is Maintained without Detectable Turnover in Oocytes Arrested for Months in Mice
Source: Curr Biol. 2016 Mar 7;26(5):678–85. doi: 10.1016/j.cub.2015.12.073 (PMC4791431; doi:10.1016/j.cub.2015.12.073)
Supplement: Document S1. Supplemental Experimental Procedures, Figures S1–S4, and Table S1 [file mmc1.pdf]

Current Biology, Volume *26*

Supplemental Information

Chromosome Cohesion Established by Rec8-Cohesin in Fetal Oocytes Is Maintained without Detectable Turnover in Oocytes Arrested for Months in Mice

Sabrina Burkhardt, Máté Borsos, Anna Szydlowska, Jonathan Godwin, Suzannah A. Williams, Paula E. Cohen, Takayuki Hirota, Mitinori Saitou, and Kikuë Tachibana-Konwalski

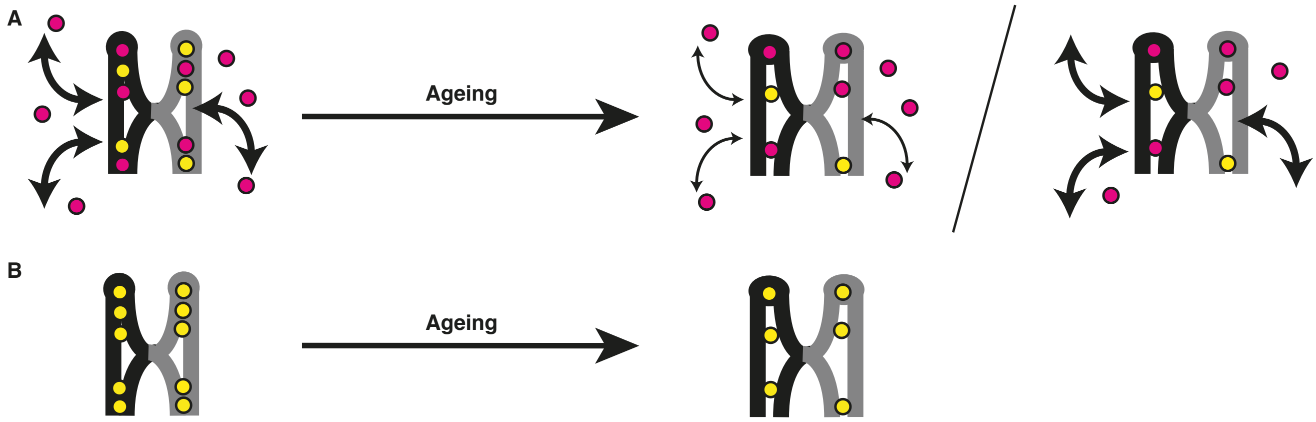

Figure S1

Figure S1. Related to Figure 1.

Model of cohesion maintenance in aging oocytes.

Models of cohesion maintenance with or without turnover to explain the decrease in chromosomal cohesin and increase in chromosome segregation errors with age. Maternal and paternal homologous chromosomes (black and grey) are held together by chiasmata mediated by cohesin distal to crossover sites.

(A) Cohesion is established during meiotic S phase (yellow) and maintained with turnover as new cohesive structures are built after S phase (red). The cohesion establishment mechanism becomes defective (black arrows) or cohesin complexes needed for replenishment (red) deteriorate in ageing oocytes.

(B) Cohesion is established during meiotic S phase (yellow) and maintained without turnover. Chromosomal cohesin decays and is irreplaceably lost from chromosomes in ageing oocytes.

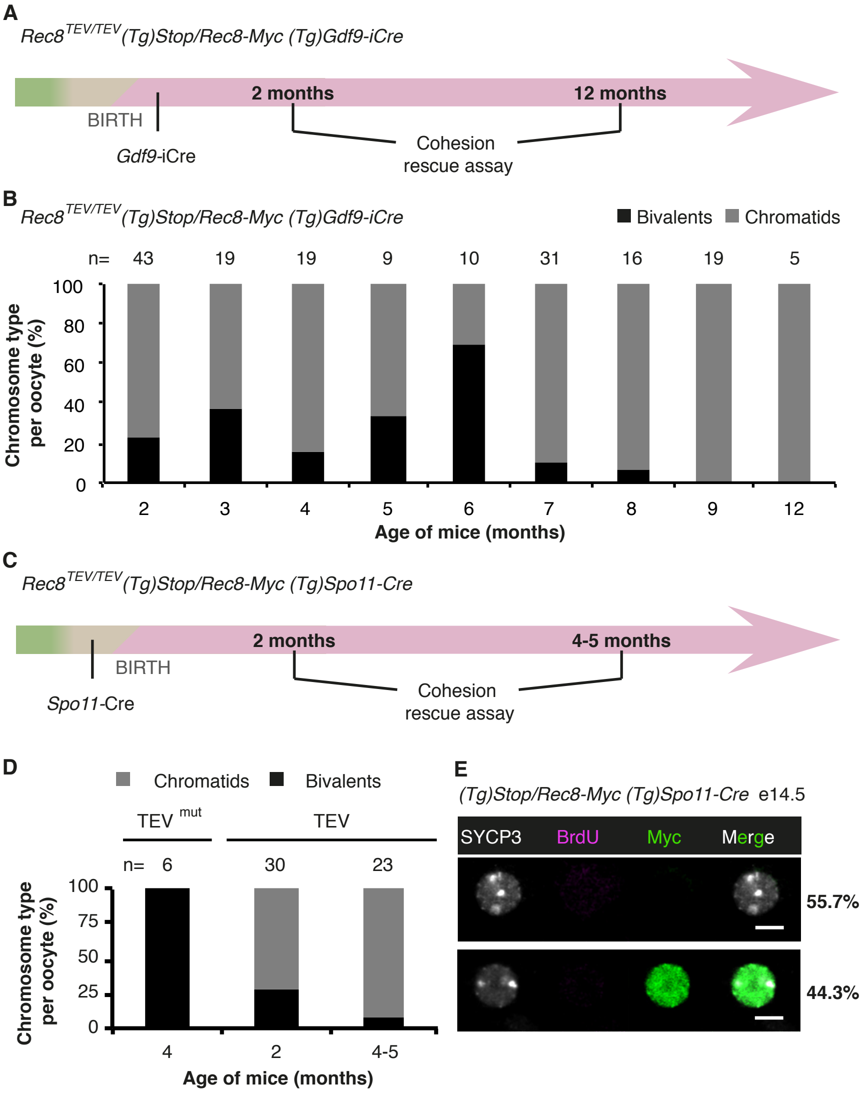
A
Rec8 TEV/TEV (Tg)Stop/Rec8-Myc (Tg)Gdf9-iCre
2 months
12 months
BIRTH
Gdf9-iCre
Cohesion rescue assay
B
Rec8 TEV/TEV (Tg)Stop/Rec8-Myc (Tg)Gdf9-iCre
Bivalents
Chromatids
n= 43 19 19 9 10 31 16 19 5
Chromosome type per oocyte (%)
100
80
60
40
20
0
2 3 4 5 6 7 8 9 12
Age of mice (months)
C
Rec8 TEV/TEV (Tg)Stop/Rec8-Myc (Tg)Spo11-Cre
2 months
4-5 months
BIRTH
Spo11-Cre
Cohesion rescue assay
D
Chromatids
Bivalents
TEV mut
TEV
n= 6 30 23
Chromosome type per oocyte (%)
100
75
50
25
0
4 2 4-5
Age of mice (months)
E
(Tg)Stop/Rec8-Myc (Tg)Spo11-Cre e14.5
SYCP3
BrdU
Myc
Merge
55.7%
44.3%

Figure S2

Figure S2. Related to Figure 2; Table S1.

***De novo* expression of Rec8-Myc using *Gdf9*-iCre or *Spo11*-Cre results in generation of cohesive structures during meiotic S phase and homologous recombination.**

(A) Timing of cohesion rescue assay utilizing *Gdf9*-iCre to activate Rec8-Myc in oocytes shortly after birth. Green, meiotic DNA replication; beige, homologous recombination; pink, dictyate stage. See also Figure 2 and Table S1 for evaluation of accurate deletion timing of *Gdf9*-iCre.

(B) Quantification of cohesion rescue assay performed with $Rec8^{TEV/TEV}$ *(Tg)Stop/Rec8-Myc (Tg)Gdf9-iCre* oocytes from females ranging from 2-12 months. The cell number for each time point is indicated (n). Oocytes were obtained from > 1 female for all time points except 8 and 12 months.

(C) Since the Spo11 endonuclease produces the DSBs that initiate homologous recombination, we considered using a Cre recombinase under the *Spo11* promoter to activate *Rec8-Myc*. Green, meiotic DNA replication; beige, homologous recombination; pink, dictyate stage. See also Figure 2 and Table S1 for evaluation of accurate deletion timing of *Spo11*-Cre.

(D) Activation of Rec8-Myc by *Spo11*-Cre results in generation of cohesive structures. Quantification of cohesion rescue assay in $Rec8^{TEV/TEV}$ *(Tg)Stop/Rec8-Myc (Tg)Spo11-Cre* oocytes. Oocytes were obtained from > 3 females for all time points using TEV protease.

(E) *Spo11*-Cre is active during meiotic recombination. e14.5 *(Tg)Stop/Rec8-Myc (Tg)Spo11-Cre* embryonic ovaries were scored for chromosomal Rec8-Myc staining in recombining (SYCP3-positive) germ cells, mean is given, SEM=1.6%, n>1000 cells from 7 females. Scale bar, 10 μm.

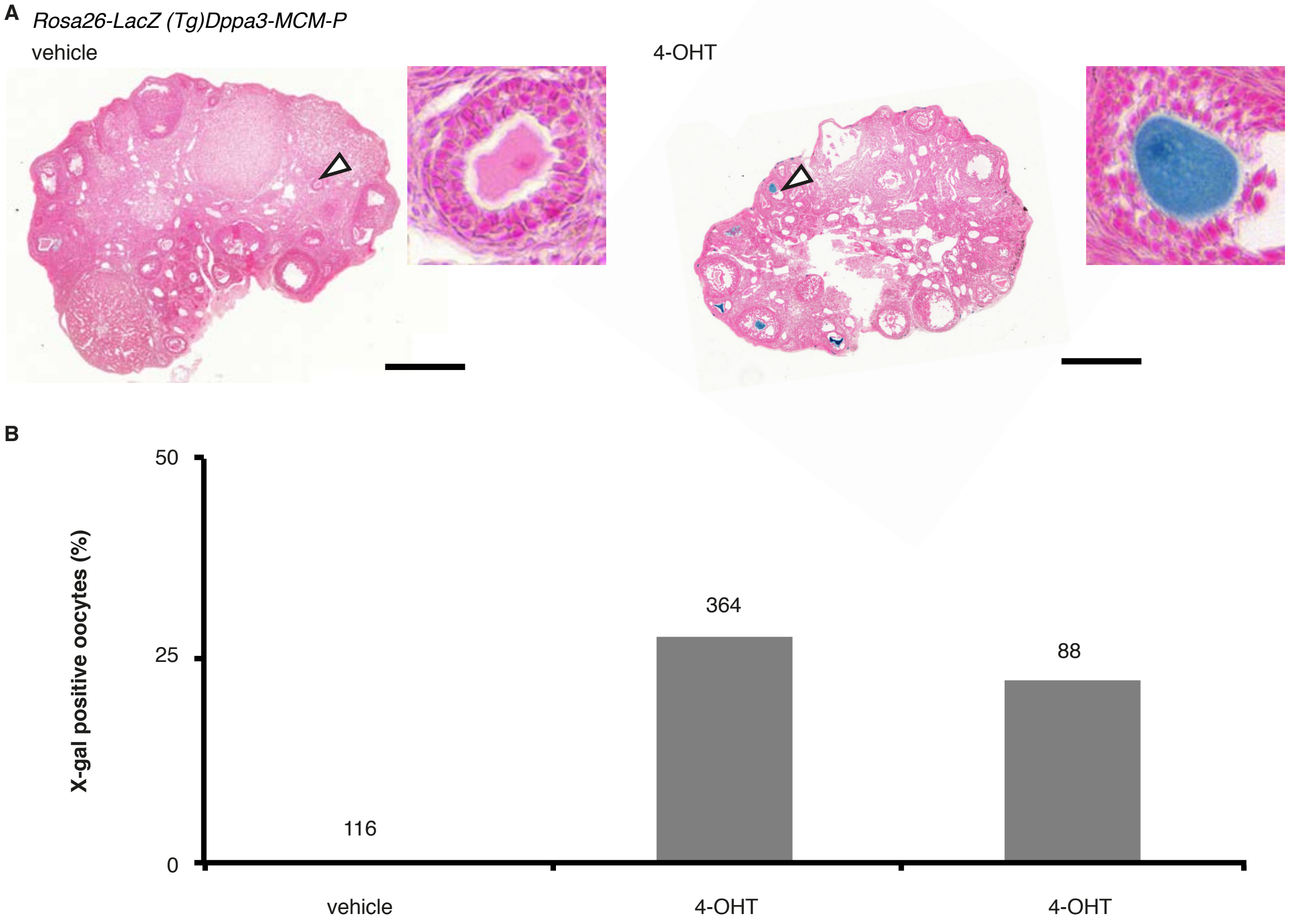
A
Rosa26-LacZ (Tg)Dppa3-MCM-P
vehicle
4-OHT
B
50
25
0
X-gal positive oocytes (%)
116
364
88
vehicle
4-OHT
4-OHT

Figure S3

Figure S3. Related to Figure 3; Figure 4; Table S1.

Oocyte-specific deletion efficiency of *(Tg)Dppa3*-MCM-P in adult ovary.

(A) Representative histological sections of individual *Rosa26-LacZ (Tg)Dppa3-MCM-P* mice after treatment with vehicle or 4-OHT. Arrowhead, magnified oocyte. Scale bar, 500 µm.

(B) Oocytes larger than 30 µm were scored. Total cell numbers are indicated.

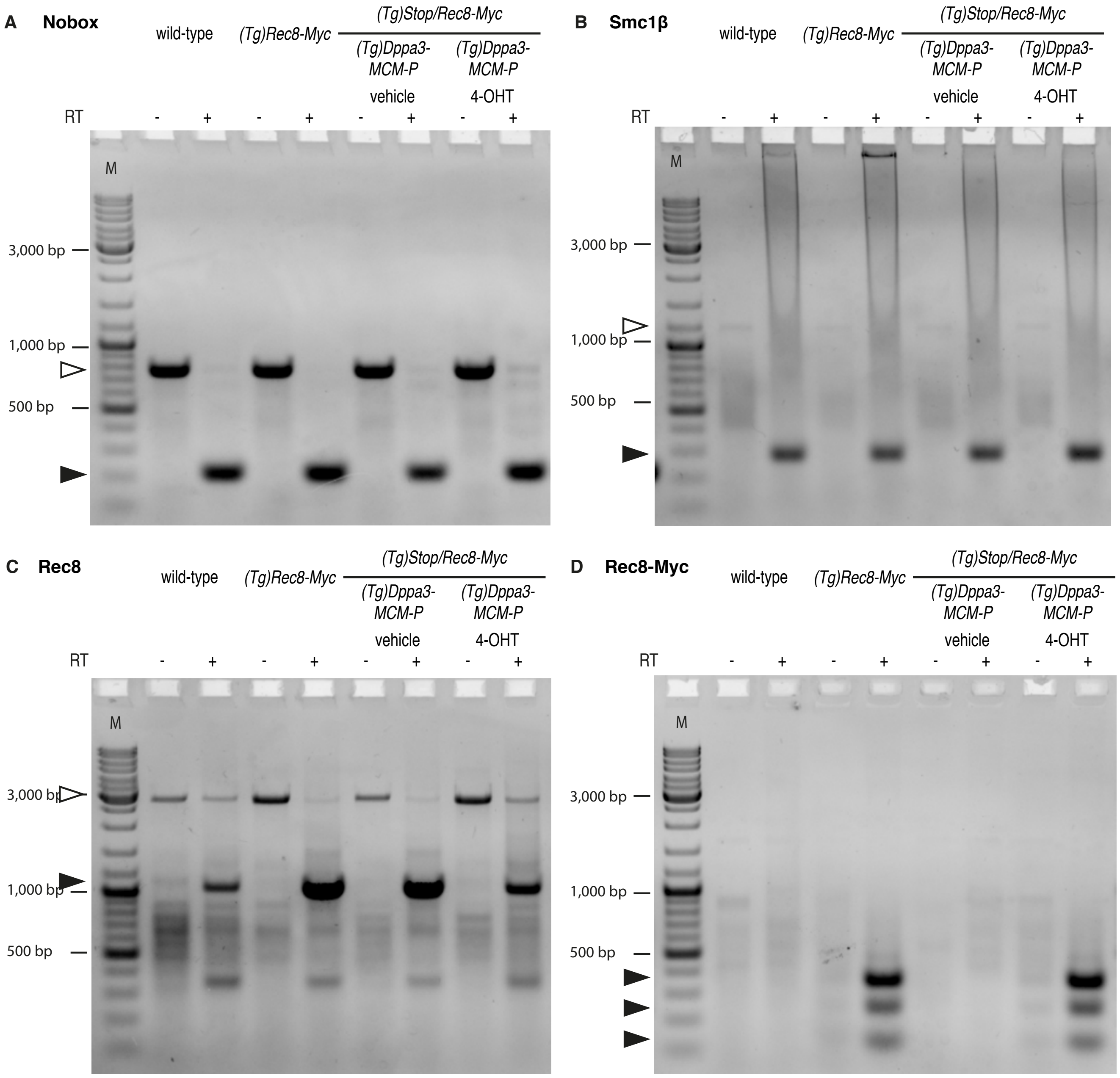
A Nobox
wild-type
(Tg)Rec8-Myc
(Tg)Stop/Rec8-Myc
(Tg)Dppa3-MCM-P
vehicle
(Tg)Dppa3-MCM-P
4-OHT
RT
- + - + - + - +
M
3,000 bp
1,000 bp
500 bp
B Smc1β
wild-type
(Tg)Rec8-Myc
(Tg)Stop/Rec8-Myc
(Tg)Dppa3-MCM-P
vehicle
(Tg)Dppa3-MCM-P
4-OHT
RT
- + - + - + - +
M
3,000 bp
1,000 bp
500 bp
C Rec8
wild-type
(Tg)Rec8-Myc
(Tg)Stop/Rec8-Myc
(Tg)Dppa3-MCM-P
vehicle
(Tg)Dppa3-MCM-P
4-OHT
RT
- + - + - + - +
M
3,000 bp
1,000 bp
500 bp
D Rec8-Myc
wild-type
(Tg)Rec8-Myc
(Tg)Stop/Rec8-Myc
(Tg)Dppa3-MCM-P
vehicle
(Tg)Dppa3-MCM-P
4-OHT
RT
- + - + - + - +
M
3,000 bp
1,000 bp
500 bp

Figure S4

Figure S4. Related to Figure 4.

Detection of Rec8-Myc transcript in adult ovary.

(A-D) Rec8-Myc mRNA is detectable in ovary of 4-OHT treated *(Tg)Stop/Rec8-Myc (Tg)Dppa3-MCM-P* mice. Entire agarose gels for mRNA detection of (A) Nobox, (B) Smc1β, (C) Rec8, and (D) Rec8-Myc by RT-PCR from ovary of wild-type, *(Tg)Rec8-Myc*, and *(Tg)Stop/Rec8-Myc (Tg)Dppa3-MCM-P* (vehicle or 4-OHT treated) mice are displayed. Black arrowhead, amplicon size for mRNA detection. White arrowhead, amplicon size for genomic DNA. RT, reverse transcriptase. M, DNA ladder.

Table S1. Related to Figure 2; Figure 3; Figure 4; Figure S2; Figure S4.

Overview of the deletion efficiency of female germline specific *(Tg)Cre* lines evaluated by up to 3 different approaches.

	**Deletion timing**		**Deletion efficiency**		
	**Developmental timing**	**Cell cycle stage**	**X-gal positive oocytes**[a]	**Genetic analysis**[b]	**Single cell PCR**[c]
(Tg)Gdf9-iCre [S1]	e13.5 *	Meiotic S phase, recombination	-	90% (20 pups)	-
(Tg)Spo11-Cre	e13.5	Meiotic S phase, recombination	94% (2♀)	100% (18 pups)	89% (53 cells/10♀)
(Tg)Dppa3-MCM-P [S2]	Inducible	Inducible	25% (2♀)	28% (46 pups)	46% (71 cells/8♀)

* High inter-cell & inter-mouse variability

[a] X-gal positive oocytes were scored in relation to unstained oocytes in adult ovary obtained from *Rosa26-LacZ* crosses to the respective *(Tg)Cre* line. Negative controls (*Rosa26-LacZ*, or vehicle treated *Rosa26-LacZ (Tg)Dppa3-MCM-P*) showed no X-gal positive staining.

[b] Deletion efficiency was determined by genotyping F1 offspring of *(Tg)Stop/Rec8-Myc (Tg)Cre* x *wild-type* crosses for deleted *Stop* cassette.

[c] Single-cell genotyping for deletion of *Stop* cassette was performed in context of live cell imaging experiments using single oocytes from *Rec8*$^{TEV/TEV}$*(Tg)Stop/Rec8-Myc (Tg)Cre* mice.

Supplemental Experimental Procedures

Mice, 4-hydroxy-tamoxifen injections, Caesarean section, genotyping

The care and use of the mice were in accordance with the guidelines of the International guiding principles for biomedical research involving animals (CIOMS, the Council for International Organizations of Medical Sciences). All of the studies were performed on a mixed BL6/129SV genetic background. Cre expression in *(Tg)Dppa3-MCM-P* mice was induced by two consecutive intraperitoneal injections within four days of 3 mg 4-OHT (Sigma, H6278) dissolved in sunflower oil (Sigma, S5007) as originally described [S2]. *Dppa3*-MCM-P expression in e10.5 embryos was induced by a single intraperitoneal injection of pregnant females with 2 mg 4-OHT. Caesarean sections on pregnant females were performed on e19.0. Once the pregnant females were killed by cervical dislocation, the abdomen was opened and each uterine horn was opened carefully with forceps. The pups and placenta were gently extruded and the amniotic sac opened. Amniotic fluid was removed by softly rubbing with a paper towel until the pups breathed. After disconnecting the umbilical cord the pups were placed with foster mothers. Post-experimental genotyping for deletion of STOP cassette in single oocytes was performed using KAPA Mouse Genotyping kit (KK7352).

In vitro culture, microinjection, and time-lapse confocal microscopy

Fully grown mouse GV oocytes were isolated, cultured, and injected as described previous [S3]. Briefly, injection of mRNA for H2B-mCherry and CenpB-EGFP was performed to monitor chromosomes and centromeres, respectively. Injection of TEV^{mut} or TEV was carried out for cohesin turnover analysis. IBMX was washed out 2 hours after injection to resume meiosis. Chromosome type in oocytes was scored as containing either 20 bivalent chromosomes or at least 72 single chromatids (and no bivalents) by 5 h post-GVBD. Imaging was performed as described previously [S3]. Briefly, a customized Zeiss LSM510 META confocal microscope equipped with P C-Apochromat 63x/1.2 NA water immersion objective lens was used for image acquisition. Chromosomes labeled with H2B-mCherry were tracked with an EMBL-developed tracking macro adapted to our microscope [S4]. Image stacks of 11 slices of 2 μm were captured every 30 min.

X-Gal staining of adult and embryonic ovaries, histology

Fixation was performed for 5 hours at 4 °C for adult, and for 20 minutes at RT for embryonic gonads in 0.2% glutaraldehyde, 2 mM $MgCl_2$ in PBS. Gonads were washed three times in wash solution (2 mM MgCl2, 0.1% sodium deoxycholate, 0.2% nonidet P-40 in 0.1 M NaPi (pH 7.4) buffer) and stained in wash solution containing (5 mM potassium ferrocyanide, 5mM potassium ferricyanide, 1 mg/ml of X-gal (Sigma, B4252) overnight at 37 °C. Next day, gonads were washed 3 times in wash solution and left in 70% ethanol until conventional histology processing. 3 μm thick sections were counterstained with Nuclear Fast Red and imaged with a Mirax Slide Scanner (Zeiss) and analyzed in Panoramic Viewer (3DHistotech).

Meiotic prophase spreading and *in situ* staining

To obtain meiotic prophase oocytes, embryonic ovaries of different aged embryos (e13.5-17.5) were isolated. For BrdU pulse labeling, pregnant females were injected IP with 50 mg/kg BrdU (Invitrogen) dissolved in embryo water (Sigma, W1503) 30 min before embryo isolation. Spreads were prepared using methods previously described [S5] with one exception: when preparing cells for DDX4 staining, in order to preserve the cytoplasmic protein fraction 2% PFA solution without 0.2% Triton-X 100 was used. Immunofluorescent staining was performed using rabbit anti-SYCP3 (Abcam, ab15093), rabbit anti-DDX4 (Abcam, ab13840), rat anti-BrdU (Abcam, ab6326), mouse anti-Myc-tag (for detection of Rec8-Myc) (Millipore, 05-724) primary antibodies, appropriate Alexa488/568/647 conjugated secondary antibodies (Invitrogen) were used for visualization, 1 μg/ml DAPI was applied for DNA counterstaining.
Imaging of spreads was performed on a Zeiss LSM780 confocal microscope equipped with 63x/1.4 plan-apochromat Oil DIC or 40x/1.4 EC plan-apochromat Oil DIC objective lenses.

Metaphase I chromosome spreads

Oocytes were washed through Tyrode's buffer (pH 2.5) at 37 °C to remove zona pellucidae. After subsequent 18 min incubation in 1:1 FBS:water at 37 °C, oocytes were fixed in a drop of 1% paraformaldehyde with 0.15% Triton X-100 and 3 mM DTT on a glass slide. After air-drying, oocytes were incubated with primary antibodies overnight at 4 °C, and then appropriate Alexa488/568/647 conjugated secondary antibodies (Invitrogen) were used for visualization, 1 µg/ml DAPI was applied for DNA counterstaining. Samples were examined on a Zeiss LSM780 confocal microscope equipped with 63x/1.4 plan-apochromat Oil DIC objective lenses. 3D surface plots of pixel intensities were generated with ImageJ.

RT-PCR

For RNA preparation, adult ovaries or testes were snapfrozen in liquid nitrogen and were grinded using glass dounce homogenizers. RNA extraction was performed with RNeasy Mini Kit (Qiagen) according to the manufactures instruction. RT reaction was carried out with SuperscriptIII (Life Technologies) using 18mer oligo dT primers (Thermo Scientific). Transcripts were detected by PCR: Rec8 (*q.v.* [S6, S7]), Nobox and Smc1β (*q.v.* [S8]), Rec8-Myc (ATCTGCTCTTGGTGCTGTCC, CAGAAATCAACTTTTGTTCACCAC).

Supplemental References

S1 Lan, Z.J., Xu, X., Cooney, A.J. (2004). Differential oocyte-specific expression of Cre recombinase activity in GDF-9-iCre, Zp3cre, and Msx2Cre transgenic mice. Biol. Reprod. *71*, 1469-1474.

S2 Hirota, T., Ohta, H., Shigeta, M., Niwa, H., Saitou, M. (2011). Drug-inducible gene recombination by the Dppa3-MER Cre MER transgene in the developmental cycle of the germ cell lineage in mice. Biol. Reprod. *85*, 367-377.

S3 Tachibana-Konwalski, K., Godwin, J., van der Weyden, L., Champion, L., Kudo, N.R., Adams, D.J., Nasmyth, K. (2010). Rec8-containing cohesin maintains bivalents without turnover during the growing phase of mouse oocytes. Genes. Dev. *24*, 2505-2516.

S4 Rabut, G., and Ellenberg, J. (2004). Automatic real-time three-dimensional cell tracking by fluorescence microscopy. J. Microsc. *216*, 131-137.

S5 Susiarjo, M., Rubio, C., Hunt, P. (2009). Analysing mammalian female meiosis. Methods Mol. Biol. *558*, 339-354.

S6 Lee, J., Yokota, T., Yamashita, M. (2002). Analyses of mRNA expression patterns of cohesin subunits Rad21 and Rec8 in mice: germ cell-specific expression of rec8 mRNA in both male and female mice. Zoolog. Sci. *19*, 539-544.

S7 Bannister, L.A., Reinholdt, L.G., Munroe, R.J., Schimenti, J.C. (2004). Positional cloning and characterization of mouse mei8, a disrupted allelle of the meiotic cohesin Rec8. Genesis *40*, 184-194.

S8 Chiang, T., Duncan, F.E., Schindler, K., Schultz, R.M., Lampson, M.A. (2010). Evidence that weakened centromere cohesion is a leading cause of age-related aneuploidy in oocytes. Curr. Biol. *20*, 1522-1528.
