# Supplementary figures and images for: Chromosome Cohesion Established by Rec8-Cohesin in Fetal Oocytes Is Maintained without Detectable Turnover in Oocytes Arrested for Months in Mice
Source: Curr Biol. 2016 Mar 7;26(5):678–85. doi: 10.1016/j.cub.2015.12.073 (PMC4791431; doi:10.1016/j.cub.2015.12.073)

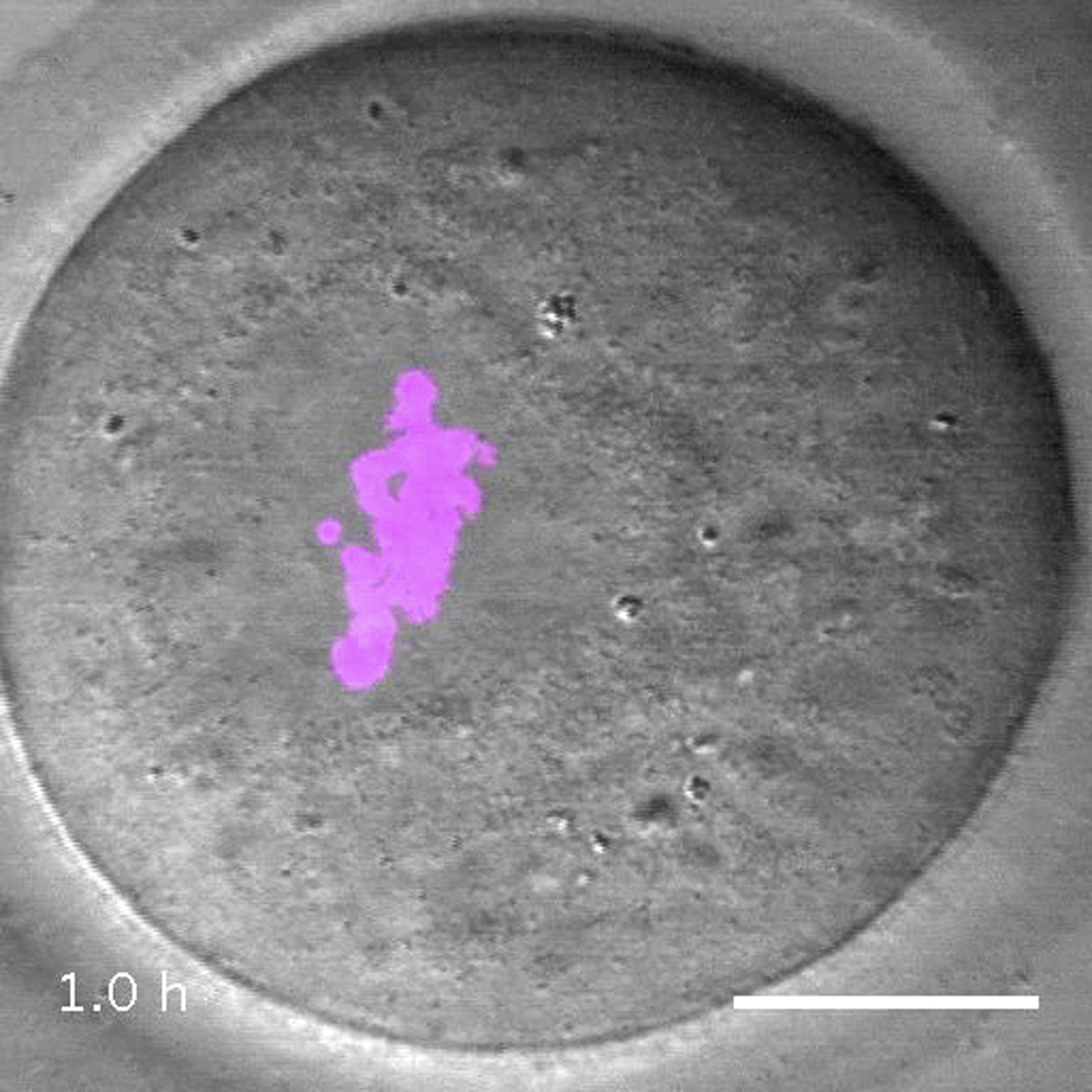

Supplement: Movie S1. Oocyte with Activated Rec8-Myc Transgene Displays Conversion of Bivalents to Chromatids by TEV Cleavage, Related to Figure 4 — Live-cell imaging of one oocyte isolated from a Rec8TEV/TEV(Tg)Stop/Rec8-Myc (Tg)Dppa3-MCM-P female (2 months post-4-OHT treatment) expressing H2B-mCherry (magenta) and TEV protease. Deletion of the Stop cassette was confirmed by single-cell genotyping post-imaging. Time intervals 0.5 hr. The scale bar represents 20 μm. [file mmc2.jpg]

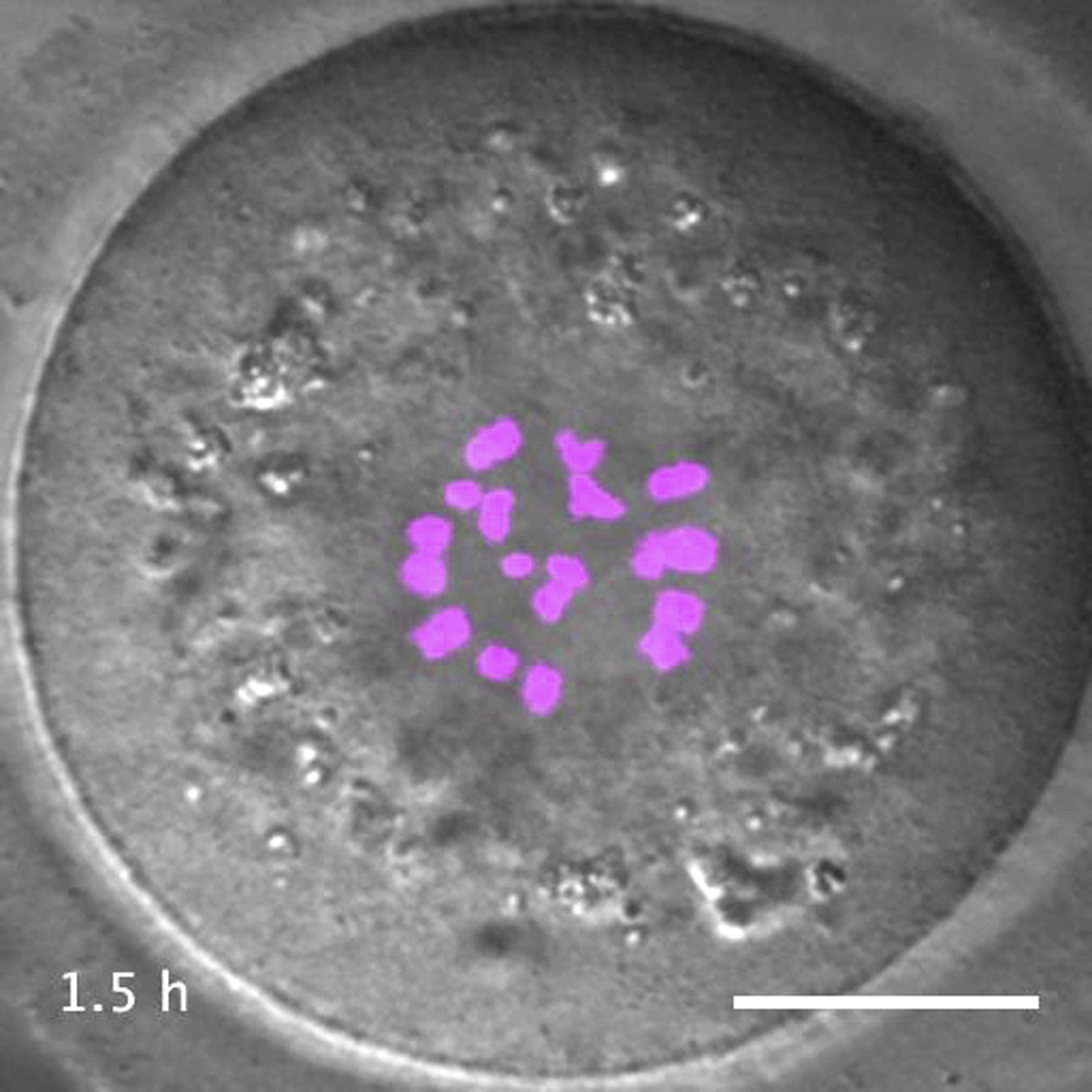

Supplement: Movie S2. Bivalents Are Retained in Oocytes When Using Mutant TEV Protease, Related to Figure 4 — Live-cell imaging of one Rec8TEV/TEV oocyte expressing H2B-mCherry (magenta) and mutant TEV protease. Time intervals 0.5 hr. The scale bar represents 20 μm. [file mmc3.jpg]
