# Supplementary material for: Chromosome Cohesion Established by Rec8-Cohesin in Fetal Oocytes Is Maintained without Detectable Turnover in Oocytes Arrested for Months in Mice
Source: Curr Biol. 2016 Mar 7;26(5):678–85. doi: 10.1016/j.cub.2015.12.073 (PMC4791431; doi:10.1016/j.cub.2015.12.073)
Supplement: Document S2. Article plus Supplemental Information [file mmc4.pdf]

Report

Current Biology

Chromosome Cohesion Established by Rec8-Cohesin in Fetal Oocytes Is Maintained without Detectable Turnover in Oocytes Arrested for Months in Mice

Graphical Abstract

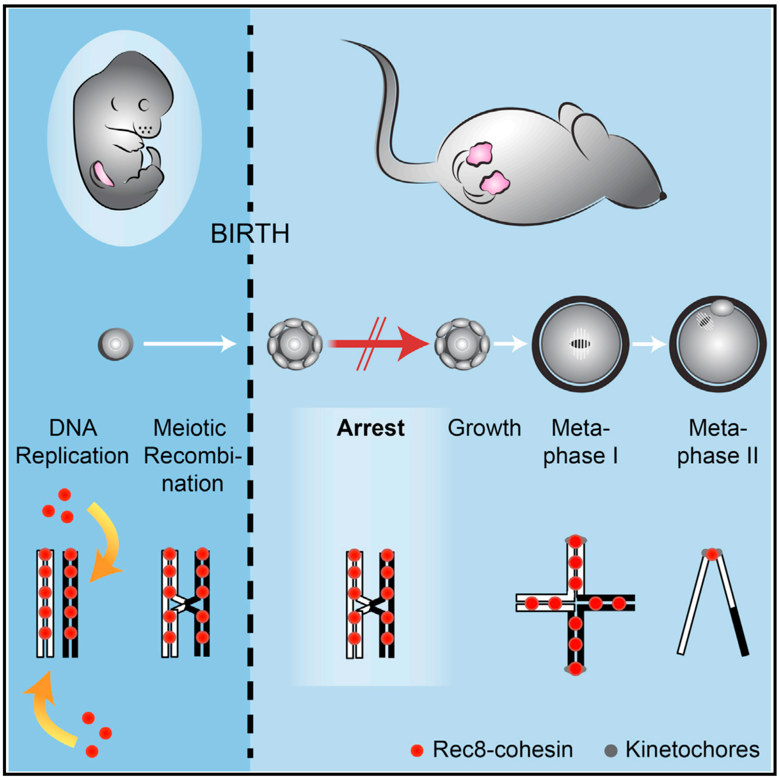

Highlights

- Rec8-mediated chromosome cohesion is established in fetal oocytes
- Bivalent cohesion is maintained without detectable turnover in arrested oocytes
- Irreversible cohesin loss underlies age-related chromosome missegregation

Authors

Sabrina Burkhardt, Máté Borsos, Anna Szydlowska, ..., Takayuki Hirota, Mitinori Saitou, Kikuë Tachibana-Konwalski

Correspondence

kikue.tachibana@imba.oeaw.ac.at

In Brief

How chromosome cohesion is maintained in female germ cells arrested for months or decades is poorly understood. Burkhardt et al. show that cohesion is built in fetal oocytes and after birth is maintained without detectable renewal for months. This implies that the oocyte's inability to renew cohesion contributes to maternal age-related trisomies.

CrossMark

Burkhardt et al., 2016, Current Biology *26*, 678–685
March 7, 2016 ©2016 The Authors
http://dx.doi.org/10.1016/j.cub.2015.12.073

CellPress

Current Biology
Report

Chromosome Cohesion Established by Rec8-Cohesin in Fetal Oocytes Is Maintained without Detectable Turnover in Oocytes Arrested for Months in Mice

Sabrina Burkhardt,[1] Máté Borsos,[1] Anna Szydlowska,[1] Jonathan Godwin,[2] Suzannah A. Williams,[3] Paula E. Cohen,[4] Takayuki Hirota,[5,6] Mitinori Saitou,[5,6] and Kikuë Tachibana-Konwalski[1,*]
[1]Institute of Molecular Biotechnology of the Austrian Academy of Sciences (IMBA), Vienna Biocenter Campus, Dr. Bohr-Gasse 3, Vienna 1030, Austria
[2]Department of Biochemistry, University of Oxford, South Parks Road, Oxford OX1 3QU, UK
[3]Nuffield Department of Obstetrics and Gynaecology, John Radcliffe Hospital, University of Oxford, Women's Centre, Level 3, Oxford OX3 9DU, UK
[4]Department of Biomedical Sciences and Center for Reproductive Genomics, Cornell University, Tower Road, Ithaca, NY 14853, USA
[5]Department of Anatomy and Cell Biology, Graduate School of Medicine, Kyoto University, Yoshida-Konoe-cho, Sakyo-ku, Kyoto 606-8501, Japan
[6]JST, ERATO, Yoshida-Konoe-cho, Sakyo-ku, Kyoto 606-8501, Japan
*Correspondence: kikue.tachibana@imba.oeaw.ac.at
http://dx.doi.org/10.1016/j.cub.2015.12.073
This is an open access article under the CC BY license (http://creativecommons.org/licenses/by/4.0/).

SUMMARY

Sister chromatid cohesion mediated by the cohesin complex is essential for chromosome segregation in mitosis and meiosis [1]. Rec8-containing cohesin, bound to Smc3/Smc1α or Smc3/Smc1β, maintains bivalent cohesion in mammalian meiosis [2–6]. In females, meiotic DNA replication and recombination occur in fetal oocytes. After birth, oocytes arrest at the prolonged dictyate stage until recruited to grow into mature oocytes that divide at ovulation. How cohesion is maintained in arrested oocytes remains a pivotal question relevant to maternal age-related aneuploidy. Hypothetically, cohesin turnover regenerates cohesion in oocytes. Evidence for post-replicative cohesion establishment mechanism exists, in yeast and invertebrates [7, 8]. In mouse fetal oocytes, cohesin loading factor Nipbl/Scc2 localizes to chromosome axes during recombination [9, 10]. Alternatively, cohesion is maintained without turnover. Consistent with this, cohesion maintenance does not require Smc1β transcription, but unlike Rec8, Smc1β is not required for establishing bivalent cohesion [11, 12]. Rec8 maintains cohesion without turnover during weeks of oocyte growth [3]. Whether the same applies to months or decades of arrest is unknown. Here, we test whether Rec8 activated in arrested mouse oocytes builds cohesion revealed by TEV cleavage and live-cell imaging. Rec8 establishes cohesion when activated during DNA replication in fetal oocytes using tamoxifen-inducible Cre. In contrast, no new cohesion is detected when Rec8 is activated in arrested oocytes by tamoxifen despite cohesin synthesis. We conclude that cohesion established in fetal oocytes is maintained for months without detectable turnover in dictyate-arrested oocytes. This implies that women's fertility depends on the longevity of cohesin proteins that established cohesion in utero.

RESULTS AND DISCUSSION

The frequency of clinically recognized trisomic pregnancies increases with maternal age [13]. Most aneuploid pregnancies arise as a consequence of chromosome segregation errors during the first meiotic division of female germ cells called oocytes, leading to aneuploid eggs [13–15]. On average, 20% of human eggs and 1%–2% of mouse eggs are aneuploid [14]. In aging human and mouse oocytes, cohesin levels decrease, centromeric cohesion weakens, and chromosome segregation errors increase [16–21]. To gain insights into age-related chromosome missegregation, we need a molecular understanding of cohesion establishment and maintenance in oocytes. A defining feature of mammalian oocytes is the prolonged arrest at the dictyate stage of prophase I that lasts for months in the mouse and decades in the human (Figure 1A). Crucially, it is not known whether bivalent cohesion is maintained with or without turnover during the arrest. If cohesion is maintained by cohesin turnover, then either the cohesion establishment mechanism deteriorates or the cohesin pool needed for replenishment diminishes in aging oocytes (Figure S1A). Alternatively, if cohesion is maintained without cohesin turnover, then cohesin loss from chromosomes is irreversible (Figure S1B). Either model is interesting and has the potential to explain what goes awry in aging oocytes, leading to the production of aneuploid fetuses.

A Functional Cohesion Rescue Assay in Meiosis I Oocytes

The entrapment of sister DNA molecules by cohesin complexes can be measured indirectly and directly using biochemical and

CellPress

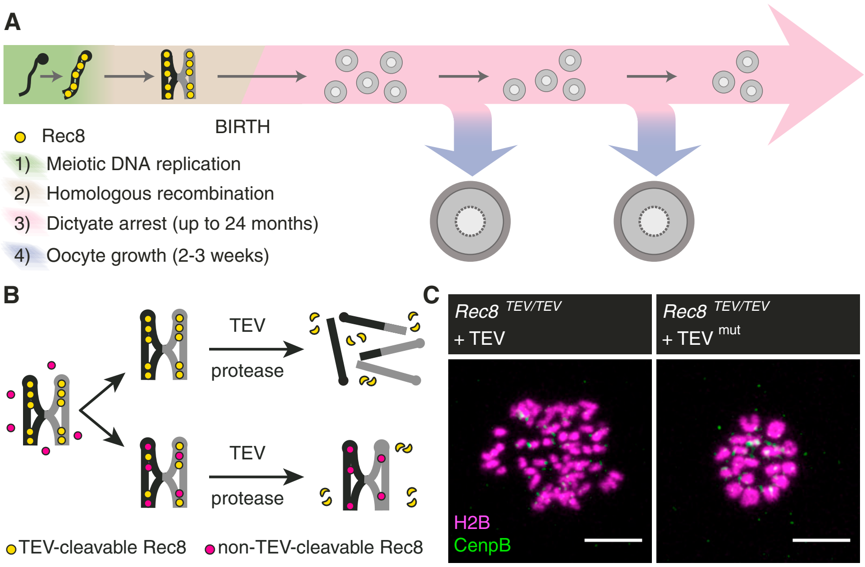

Figure 1. Probing Cohesion Maintenance during Mammalian Female Meiosis Using a Functional Cohesion Assay

(A) Mammalian female meiosis can be structured into four stages: (1) meiotic DNA replication in which sister chromatid cohesion is presumably established, (2) meiotic recombination in which reciprocal recombination of homologous chromosomes (black and gray) results in crossovers that manifest as chiasmata, (3) the prolonged resting state at the dictyate stage of prophase I after birth, and (4) the growing phase that starts when an oocyte is recruited from the resting pool and leads to formation of a mature oocyte. The duration of the dictyate arrest and growing phase correspond to mouse. Schematic is not drawn to scale.

(B) Schematic of cohesion rescue assay. TEV-cleavable Rec8 (yellow) establishes and maintains bivalent cohesion. Non-TEV-cleavable Rec8 (red) is activated after meiotic cohesion establishment. Cleavage by TEV protease reveals whether de novo expressed non-TEV-cleavable Rec8 is able to build functional cohesive structures: cleavage of bivalents into single chromatids indicates no functional loading, while resistance to TEV cleavage reveals functional cohesive structures entrapping sister DNA molecules.

(C) *Rec8*$^{TEV/TEV}$ oocytes are microinjected with mRNA encoding H2B-mCherry, CenpB-EGFP, and TEV protease. Confocal time-lapse microscopy allows scoring of chromosome type at metaphase I (5 hr post-GVBD). TEV protease efficiently converts bivalents to chromatids, which are detected as at least 72 single chromatids and no bivalents (oocytes analyzed n = 40), while no cleavage of all 20 bivalents is observed using mutant TEV protease (TEV^{mut}; oocytes analyzed n = 16). Scale bar, 10 μm.

See also Figure S1.

cell biological approaches [7, 22–24]. To determine whether cohesion is maintained with or without building additional cohesive structures after DNA replication, we used a functional cohesion assay that we had established previously (Figure 1B) [3]. Briefly, endogenous Rec8 contains engineered Tobacco Etch Virus (TEV) recognition sites rendering cohesin cleavable by TEV protease. TEV protease expression in *Rec8*$^{TEV/TEV}$ oocytes converts 100% of bivalents to chromatids. To test whether new cohesion is built, Rec8-Myc that is not cleavable by TEV protease is induced in addition to endogenous Rec8TEV that establishes cohesion during DNA replication (Figure 1B). If Rec8-Myc becomes incorporated into cohesin complexes that establish cohesion, then bivalents become resistant to destruction by TEV protease. If no new cohesion is established, then TEV cleavage of Rec8 converts bivalents to chromatids. We assume that over time slow cohesin decay takes place and contemporaneously occurring reloading of Rec8-Myc can be revealed by TEV cleavage to rapidly destroy endogenous Rec8. The genetic components are *Rec8*$^{TEV/TEV}$ oocytes that also contain a conditional silent *BAC* transgene with a *Stop* cassette flanked by *LoxP* sites, *(Tg)Stop/Rec8-Myc*. Cre recombinase deletes the *Stop* cassette and activates *Rec8-Myc* transgene expression. Deletion of the *Stop* cassette using *(Tg)Sox2*-Cre in the early embryo, before primordial germ cell specification, rescues bivalent cohesion in mature oocytes [3, 25]. Therefore, Rec8-Myc is capable of establishing functional cohesion when activated before meiosis.

To visualize the cohesion status of chromosomes in meiosis I oocytes, we used a microinjection and live-cell imaging approach. Mature germinal vesicle (GV) stage oocytes isolated from sexually mature females are cultured in 3-isobutyl-1-methylxanthine (IBMX)-containing medium to inhibit germinal vesicle breakdown (GVBD). GV oocytes are microinjected with mRNA encoding H2B-mCherry to visualize chromosomes, TEV protease and another marker such as CenpB-EGFP that localizes to kinetochores. After expression of the mRNA constructs, oocytes are released into IBMX-free medium to resume meiosis and followed by confocal time-lapse microscopy. Bivalents are converted to chromatids within 3–4 hr in *Rec8*$^{TEV/TEV}$ oocytes expressing wild-type but not mutant TEV protease (TEV^{mut}), demonstrating that bivalent cohesion is intact without TEV cleavage (Figure 1C). Therefore, the comparison of bivalents versus chromatids enables the visualization of functional cohesion in live oocytes.

Gdf9-iCre and *Spo11*-Cre Delete during Meiotic DNA Replication

To test whether cohesion is maintained with or without turnover during the dictyate arrest, we sought to activate the *Rec8-Myc* transgene in arrested oocytes shortly after birth. To achieve this, we selected the widely used deleter strain *(Tg)Gdf9-iCre* in which Cre expression is controlled by the promoter of growth differentiation factor 9 (Gdf9) [11, 26–34]. *(Tg)Gdf9-iCre* is thought to delete conditional alleles in oocytes shortly after birth, i.e., days after DNA replication and meiotic recombination (Figure 2A).

Since it is important that *Rec8-Myc* transgene activation occurs after meiotic DNA replication, we carried out due diligence to confirm that *Gdf9*-iCre does not delete during DNA replication. Using a conditional LacZ reporter strain (*Rosa26-LacZ*) [35], we analyzed *Rosa26-LacZ (Tg)Gdf9-iCre* ovaries on embryonic day E13.5 when oocytes enter meiosis. Unexpectedly, seven out of ten fetal ovaries contained X-gal positive cells (Figure 2B), suggesting that deletion might occur as

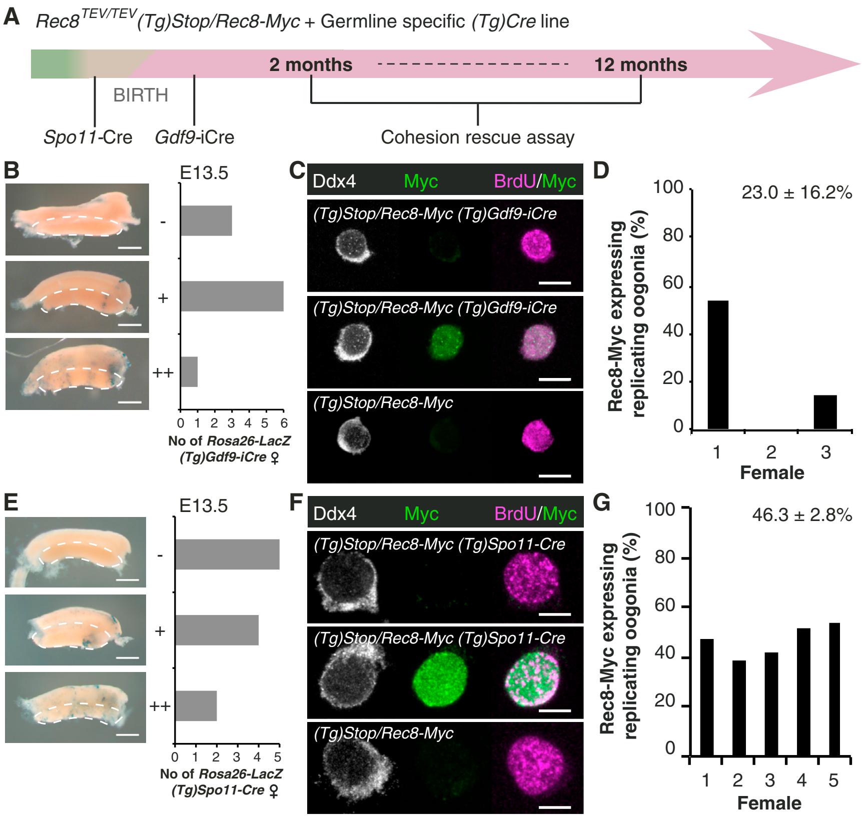

Figure 2. *Gdf9*-iCre and *Spo11*-Cre Activate Rec8-Myc during Meiotic DNA Replication

(A) Interpretation of the cohesion rescue assay requires activation of the *Rec8-Myc* transgene after meiotic DNA replication (green). Thus, *Gdf9*-iCre or *Spo11*-Cre was used to activate Rec8-Myc in oocytes shortly after birth (dictyate stage, pink) or during homologous recombination (beige), respectively.

(B) Timely deletion analysis of *Gdf9*-iCre. Scoring of X-gal positive cells in *Rosa26-LacZ (Tg)Gdf9-iCre* embryonic ovaries at E13.5 according to classification into negative (–), weakly positive (+), and positive (++); n = 10 female embryos. The dashed line indicates the gonad. Scale bar, 1 mm.

(C and D) *Gdf9*-iCre activates Rec8-Myc during meiotic S phase. Embryonic day E13.5 *(Tg)Stop/Rec8-Myc (Tg)Gdf9-iCre* embryonic ovaries were scored for Rec8-Myc expressing replicating (BrdU-positive) oogonia identified by germ cell-specific cytoplasmic Ddx4 staining; n = 3 females. No Myc signal was observed in oogonia from control *(Tg)Stop/Rec8-Myc* females; n = 2 females.

(C) Representative images; scale bar, 10 μm.

(D) Quantification for *(Tg)Stop/Rec8-Myc (Tg) Gdf9-iCre* embryonic ovaries; n > 100 cells per female. Mean ± SEM given.

(E–G) Timely deletion analysis of *Spo11*-Cre.

(E) Scoring of X-gal positive cells in *Rosa26-LacZ (Tg)Spo11-Cre* embryonic ovaries at E13.5 according classification into negative (–), weakly positive (+), and positive (++); n = 11 female embryos. The dashed line indicates the gonad. Scale bar, 1 mm.

(F and G) *Spo11*-Cre activates Rec8-Myc during meiotic S phase. Embryonic day E14.5 *(Tg)Stop/Rec8-Myc (Tg)Spo11-Cre* embryonic ovaries were scored for Rec8-Myc expressing replicating (BrdU-positive) oogonia identified by germ cell-specific cytoplasmic Ddx4 staining; n = 5 females. No Myc signal was observed in oogonia from control *(Tg)Stop/Rec8-Myc* females; n = 3 females.

(F) Representative images; scale bar, 10 μm.

(G) Quantification for *(Tg)Stop/Rec8-Myc (Tg)Spo11-Cre* embryonic ovaries; n > 500 cells per female. Mean ± SEM given.

See also Figure S2 and Table S1.

early as meiotic DNA replication. Indeed, Rec8-Myc is expressed in up to 50% of replicating germ cells, identified as BrdU- and Ddx4-positive cells, in oocytes from *(Tg)Stop/Rec8-Myc (Tg)Gdf9-iCre* females (Figures 2C and 2D). *Gdf9*-iCre activated Rec8-Myc before or during meiotic DNA replication in two out of three female embryos (Figure 2D). Overall *Gdf9*-iCre deletes with high efficiency (Table S1), but the deletion timing varies between mice and between oocytes within one mouse. In agreement with this, cohesion rescue experiments using oocytes from *Rec8*$^{TEV/TEV}$ *(Tg)Stop/Rec8-Myc (Tg)Gdf9-iCre* females resulted in variable rescue efficiencies (Figures S2A and S2B). It is not possible to know whether cohesion rescue in these cells is due to cohesion establishment during DNA replication or thereafter. On a technical note, while it cannot be excluded that mouse strain background and genetic locus might have some effect on the timing of Cre-mediated deletion, our analyses using two different target loci showing earlier deletion than previously thought raise concerns about the suitability of *Gdf9*-iCre for cell cycle phase-specific deletion studies.

A second approach to activate the *Rec8-Myc* transgene after meiotic DNA replication might rely on Cre recombinase controlled by a promoter driving expression of a protein required for recombination. The topoisomerase-like enzyme Spo11 generates DNA double-strand breaks that initiate recombination [36]. Therefore, we chose to test *Spo11*-Cre. If *Spo11*-Cre deletes after DNA replication and before or during recombination, then this system will test whether cohesion is built at all after DNA replication. It would not be possible to distinguish whether cohesion is generated during recombination or the dictyate-stage arrest.

To characterize the timing of *Spo11*-Cre, we used *Rosa26-LacZ* and *(Tg)Stop/Rec8-Myc* mice (Figures 2E–2G). *Spo11*-Cre also deletes during meiotic DNA replication in 46% of replicating oocytes (Figure 2G). In agreement with this, cohesion rescue is detected in some oocytes from *Rec8*$^{TEV/TEV}$ *(Tg)Stop/Rec8-Myc Spo11*-Cre females (Figures S2C and S2D). We conclude that neither *Gdf9*-iCre nor *Spo11*-Cre allow consistent activation of Rec8-Myc after meiotic DNA replication in fetal oocytes.

Timely Controlled Rec8 Activation in Fetal and Adult Arrested Oocytes

To demonstrate whether arrested oocytes maintain cohesion with or without turnover, it is important to activate *Rec8-Myc* transgene expression after meiotic DNA replication and homologous recombination. Since there are currently no mouse strains other than *(Tg)*

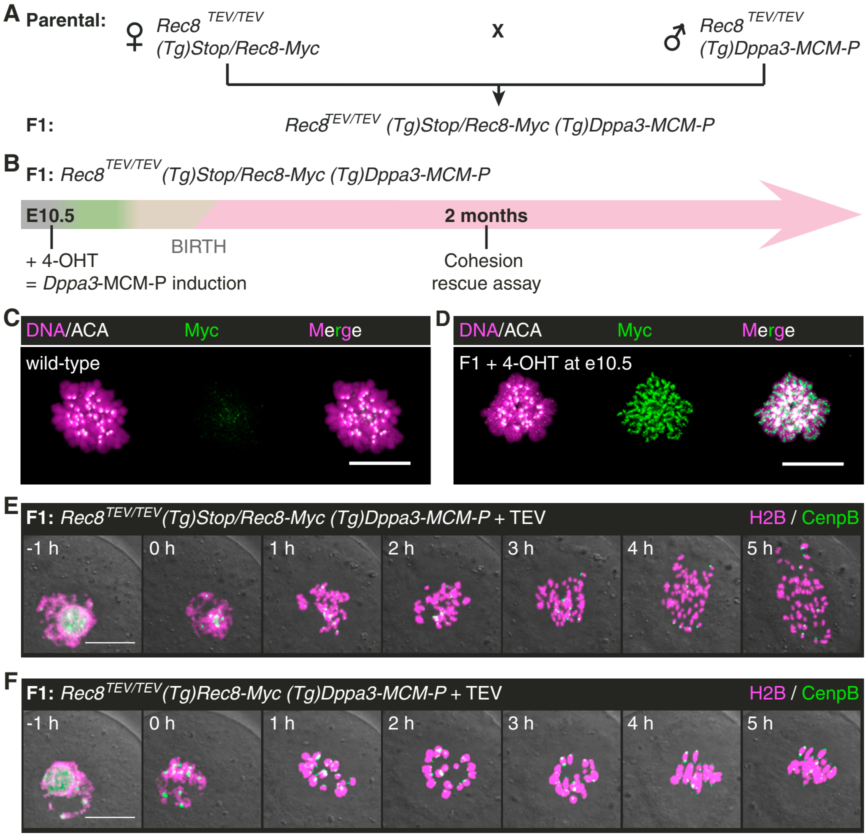

Figure 3. Rec8 Activated in Fetal Oocytes Builds Cohesive Structures

(A) Mating scheme to obtain female F1 offspring of the required genotype *Rec8$^{TEV/TEV}$ (Tg)Stop/Rec8-Myc (Tg)Dppa3-MCM-P*.

(B) Activation of Rec8-Myc during meiotic S phase in fetal oocytes. Pregnant *Rec8$^{TEV/TEV}$ (Tg)Stop/Rec8-Myc* females are injected with 4-OHT on embryonic day E10.5 to induce deletion by *Dppa3*-MCM-P in embryos. Oocytes are isolated from F1 females with the appropriate genotype. Green, meiotic DNA replication; beige, homologous recombination; pink, dictyate stage.

(C and D) Metaphase I chromosome spread showing localization of Rec8-Myc to bivalent chromosomes in oocytes from (C) wild-type and (D) F1 *Rec8$^{TEV/TEV}$ (Tg)Stop/Rec8-Myc (Tg)Dppa3-MCM-P* female; oocytes analyzed n = 9 and n = 7, respectively. Centromeres are marked by anti-centromere antibody (ACA). Scale bar, 20 μm.

(E and F) Representative still images of oocytes isolated from F1 *Rec8$^{TEV/TEV}$ (Tg)Stop/Rec8-Myc (Tg)Dppa3-MCM-P* females microinjected with mRNA encoding H2B-mCherry, CenpB-EGFP, and TEV protease.

(E) Bivalents are converted to chromatids in cells without *Stop* cassette deletion; n = 17 oocytes.

(F) Bivalents are retained in oocytes with successful deletion of the *Stop* cassette; n = 5 oocytes. Genotype of single cells was confirmed after live-cell imaging. Oocytes were obtained from >3 females. Scale bar, 20 μm.

See also Figure S3 and Table S1.

Gdf9-iCre that are thought to delete in arrested oocytes before growth, we chose to directly control the timing of Cre-mediated deletion by injection of 4-hydroxytamoxifen (4-OHT). Specifically, the germ cell-specific *Dppa3* promoter drives expression of Cre fused to mouse estrogen receptors (MERCreMER) and a PEST degradation motif in *(Tg)Dppa3-MCM-P* mice [37] (Table S1). MERCreMER is cytoplasmic, and 4-OHT binding to the receptors triggers translocation of the Cre-fusion to the nucleus, facilitating timely genetic deletion [38].

The challenges with this approach are a risk of deletion without 4-OHT, inefficient deletion with 4-OHT, and effects of 4-OHT on fertility. Since background deletion could result in a false positive cohesion rescue, we first tested whether there is any deletion without 4-OHT. Reassuringly, vehicle injection into *Rosa26-LacZ (Tg)Dppa3-MCM-P* females resulted in no X-gal positive oocytes in ovary sections, consistent with the negligible background reported by others (Figure S3). On the other hand, 4-OHT injection into *Rosa26-LacZ (Tg)Dppa3-MCM-P* females resulted in ~25% X-gal positive oocytes, indicating that Cre-mediated deletion had occurred (Figure S3). Since the deletion efficiency is low, oocytes with a deleted *Stop* cassette in *Rec8-Myc* will be identified by single-cell PCR genotyping after the cohesion rescue assay (Table S1). Lastly, 4-OHT-injected *(Tg)Stop/Rec8-Myc (Tg)Dppa3-MCM-P* females are fertile for up to 12 litters, and 28% of offspring inherited *(Tg)Rec8-Myc* (n = 3 females + 4-OHT; n = 3 females + vehicle). Therefore, 4-OHT and Cre-mediated deletion do not grossly affect oocyte maturation and female fertility.

To test whether 4-OHT induces sufficient levels of Rec8 to rescue bivalent cohesion in meiosis I oocytes, we embarked on a novel fetal-to-adult oocyte experiment. The idea is to activate Rec8 in fetal oocytes and perform the cohesion rescue assay on the same oocytes isolated from the adult female. Pregnant *Rec8$^{TEV/TEV}$ (Tg)Stop/Rec8-Myc* females mated to *Rec8$^{TEV/TEV}$ (Tg)Dppa3-MCM-P* males were injected with 4-OHT on embryonic day E10.5 to activate Rec8-Myc in replicating oocytes of female embryos (F1 generation) (Figure 3A). *Rec8$^{TEV/TEV}$ (Tg)Stop/Rec8-Myc (Tg)Dppa3-MCM-P* F1 females were sacrificed at 2 months for oocyte isolation (Figure 3B). If Rec8-Myc activated by 4-OHT established cohesion in a replicating fetal oocyte, then the same oocyte isolated from the adult F1 female would be expected to express Rec8-Myc. Indeed, Rec8-Myc localized to bivalents on a meiosis I chromosome spread from an oocyte isolated from a *Rec8$^{TEV/TEV}$ (Tg)Stop/Rec8-Myc (Tg)Dppa3-MCM-P* F1 female (Figures 3C and 3D). The localization of cohesin to the inter-chromatid axis of bivalents suggests, but does not demonstrate, that cohesin is entrapping sister chromatids. To test for functional cohesion, we injected oocytes with TEV protease and imaged them. Indeed, bivalent cohesion is rescued in oocytes from *Rec8$^{TEV/TEV}$ (Tg)Stop/Rec8-Myc (Tg)Dppa3-MCM-P* F1 females with a deleted *Stop* cassette (Figures 3E and 3F). This implies that sufficient levels of Rec8 are synthesized due to 4-OHT to establish cohesion and rescue bivalent cohesion in adult oocytes, at least when Rec8 is activated in fetal oocytes.

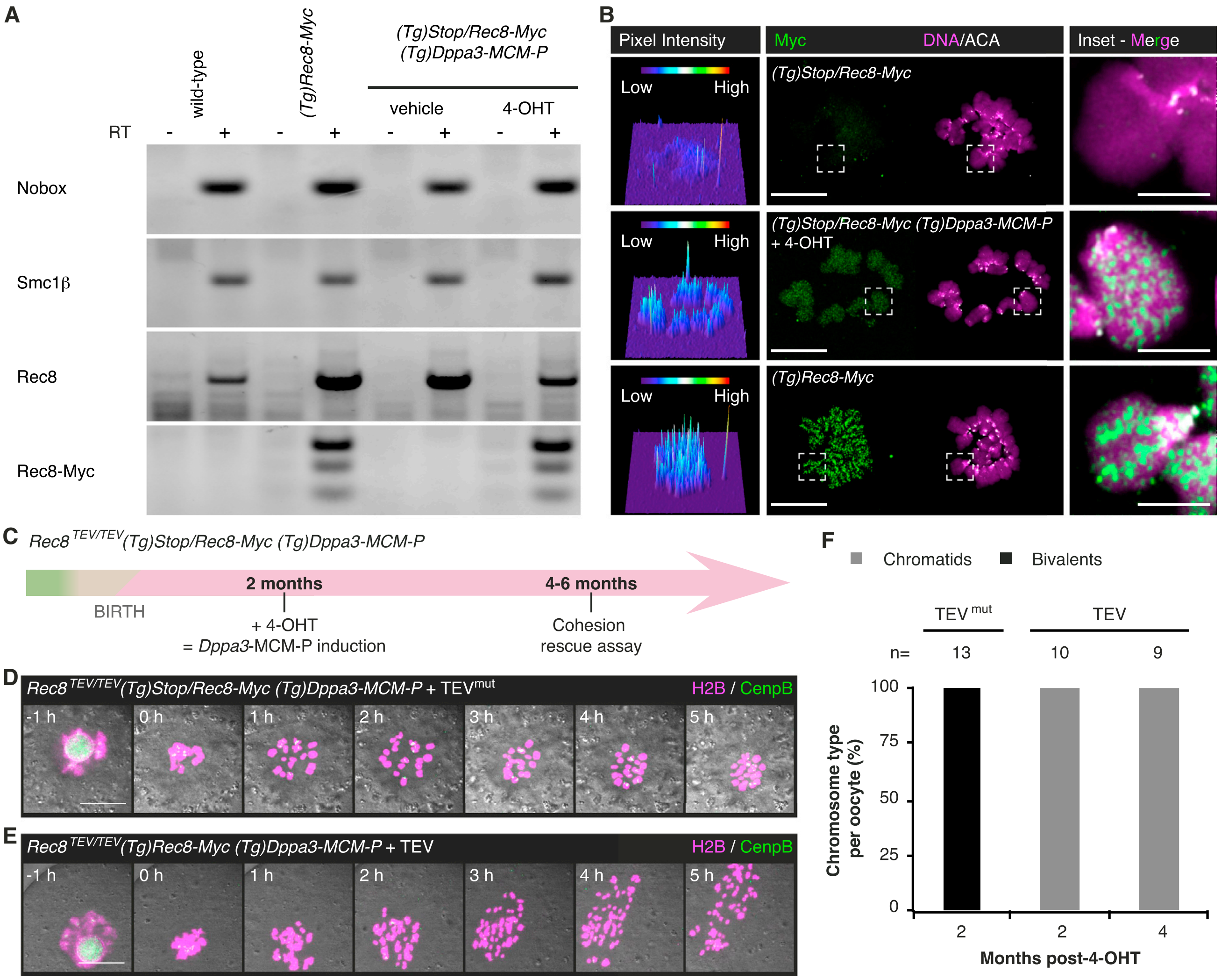

Figure 4. Bivalent Cohesion Is Maintained without Detectable Turnover of Rec8-Cohesive Structures for Months during the Dictyate Arrest

(A) Rec8-Myc is transcribed in adult ovary. Detection of mRNA for Nobox, Smc1β, Rec8, and Rec8-Myc by RT-PCR from adult ovary. RT, reverse transcriptase. See also Figure S4.

(B) Rec8-Myc is synthesized in oocytes from adult *Rec8$^{TEV/TEV}$ (Tg)Stop/Rec8-Myc (Tg)Dppa3-MCM-P* female analyzed 2 months post-4-OHT. Metaphase I chromosome spread showing expression and localization of Rec8-Myc to bivalent chromosomes. Left panel shows 3D surface plots of Rec8-Myc pixel fluorescence intensities. Centromeres are marked by ACA. Oocytes analyzed from top to bottom: n = 11, 7, 16. Inset has been brightness and contrast enhanced equally in all three panels. Scale bar, 20 μm; inset scale bar, 5 μm.

(C) Timing of cohesion rescue assay utilizing *Dppa3*-MCM-P to activate Rec8-Myc in adult female mice. Green, meiotic DNA replication; beige, homologous recombination; pink, dictyate stage.

(D and E) Representative still images of oocytes isolated from *Rec8$^{TEV/TEV}$ (Tg)Stop/Rec8-Myc (Tg)Dppa3-MCM-P* females microinjected with mRNA encoding H2B-mCherry, CenpB-EGFP, and TEV protease.

(D) Bivalents are retained in oocytes microinjected with mutant TEV mRNA.

(E) TEV protease converts bivalents to chromatids in cells with successful *Stop* cassette deletion. Single-cell genotyping was performed after live-cell imaging. Scale bar, 20 μm.

(F) Quantification of cohesion rescue assay in oocytes from *Rec8$^{TEV/TEV}$ (Tg)Stop/Rec8-Myc (Tg)Dppa3-MCM-P* females with successful *Stop* cassette deletion used 2 or 4 months post-4-OHT treatment. Oocytes were obtained from >3 females; n = number of oocytes analyzed.

See also Figure S3; Table S1; Movies S1 and S2.

Before using the timely controlled activation of Rec8 in dictyate-arrested oocytes, it was necessary to test whether Rec8 is transcribed in arrested oocytes. Rec8 transcripts are detectable in adult ovaries [39] (Figures 4A and S4), but these could be stored mRNA that was synthesized in early meiosis. To test whether Rec8-Myc is de novo transcribed in post-recombination oocytes, we injected *(Tg)Stop/Rec8-Myc (Tg)Dppa3-MCM-P* adult females with vehicle or 4-OHT and performed RT-PCR on whole ovaries. *(Tg)Rec8-Myc* ovaries served as a positive control to identify Rec8-Myc mRNA; the presence of oocytes was confirmed using the germ cell-specific transcripts Nobox and Smc1β. Rec8-Myc was expressed

specifically in 4-OHT- and not in vehicle-injected *(Tg)Stop/Rec8-Myc (Tg)Dppa3-MCM-P* ovaries (Figures 4A and S4). Since *Rec8-Myc* is under control of the endogenous promoter in the *BAC*, de novo transcription of Rec8-Myc suggests that endogenous Rec8 is also transcribed in adult oocytes.

We next tested whether Rec8 protein is synthesized in adult oocytes. While no Rec8-Myc signal was detectable on chromosome spreads from control *(Tg)Stop/Rec8-Myc* oocytes, little but detectable Rec8-Myc localized to chromosomes from oocytes isolated from *(Tg)Stop/Rec8-Myc (Tg)Dppa3-MCM-P* females injected with 4-OHT (Figure 4B). We conclude that Rec8 protein is synthesized de novo and associates with chromosomes in adult oocytes.

In the key experiment, we asked whether cohesion is maintained with or without turnover in oocytes arrested for months at the dictyate stage of prophase I. *Rec8*$^{TEV/TEV}$ *(Tg)Stop/Rec8-Myc (Tg)Dppa3-MCM-P* adult females were injected with 4-OHT to activate Rec8-Myc in oocytes, and the cohesion rescue assay was performed 2 or 4 months after activation (Figure 4C). Following TEV protease injection and time-lapse imaging, single-cell PCR identified 46% of oocytes with activated Rec8-Myc (n = 71 oocytes, 8 females, Table S1). Importantly, 100% of oocytes with activated Rec8-Myc both after 2 or 4 months displayed chromatids in meiosis I (Figures 4D–4F; Movies S1 and S2). Therefore, cohesion is maintained without turnover using newly synthesized Rec8 in oocytes arrested for several months at the dictyate stage of prophase I. Current experiments do not allow us to exclude that Rec8-cohesin complexes assembled early in meiosis might turn over. In summary, we conclude that cohesive structures maintaining bivalent cohesion must have been built before *(Tg)Dppa3*-MCM-P activation, most likely during DNA replication. Since genetic tools like *Spo11*-Cre cannot distinguish temporally between meiotic DNA replication and homologous recombination (Figures 2F, 2G, and S2E), it remains an open question whether additional cohesive structures are built during meiotic recombination or whether cohesion is established exclusively during DNA replication.

Conclusions

Overall our results show that sister chromatid cohesion mediated by Rec8-containing cohesin complexes is established in fetal oocytes and maintained without detectable turnover after birth, both during the prolonged dictyate-stage arrest and the weeks of oocyte growth [3]. To fully understand cohesin dynamics in female meiosis will require an integrated approach including other cohesin complexes and a variety of techniques. We have employed a functional cohesion rescue assay that overcomes some limitations of cohesin detection by indirect immunofluorescent staining of chromosomes, which could reflect any mode of association, e.g., binding to chromatids (non-cohesive) or holding sister chromatids together (cohesive). Our results are not mutually exclusive with the findings that the cohesin loading factor Nipbl/Scc2 localizes to chromosome axes during meiotic recombination since we have investigated post-recombination, dictyate-stage arrested oocytes [9, 10]. Moreover, it is conceivable that Nipbl/Scc2 loads different types of cohesin complexes, which may contain Rad21L rather than Rec8, onto chromosomes. Thus, it remains an open question whether cohesion is built during meiotic recombination.

The advantage of the TEV cleavage assay of cohesin combined with an inducible transgenic rescue construct is that cohesion of sister chromatids is revealed in live cells. At the same time, it is challenging to empirically determine the sensitivity of the assay toward newly built cohesion. Certainly 50% turnover is robustly detected as bivalents remain intact following TEV protease expression in *Rec8*$^{TEV/+}$ oocytes [3]. Given that as little as 13% of cohesin is sufficient for cohesion in yeast [40], it is likely that relatively few cohesin molecules mediating cohesion would be sufficient for rescue of bivalent chromosomes. It is therefore noteworthy that Rec8-Myc expression is controlled by the endogenous promoter on the *BAC*, and we have investigated the mechanism of cohesion maintenance relevant to the wild-type.

The discovery that bivalent cohesion is established predominantly, if not exclusively, in fetal oocytes has important implications for aging oocytes with increasing chromosome segregation errors [17–21]. Rather than invoking deterioration of cohesion establishment mechanisms or diminishing soluble cohesin proteins, our results suggest that age-related chromosome missegregation is due to the irreplaceable loss of cohesin complexes holding chromosomes together (Figure S1B). How cohesion is maintained at a mechanistic level for a long time and whether new cohesion is actively prevented by anti-establishment factors such as Wapl remain key questions for the future. Our work in mouse female meiosis supports the hypothesis that the inability of oocytes to build cohesion during the dictyate arrest that lasts for months or decades contributes to maternal age-related chromosome missegregation and production of aneuploid fetuses.

For material and methods, see Supplemental Information. The usage of mice followed the international guiding principles for biomedical research involving animals (the Council for International Organizations of Medical Sciences) and were in agreement with the authorizing committee.

SUPPLEMENTAL INFORMATION

Supplemental Information includes Supplemental Experimental Procedures, four figures, one table, and two movies and can be found with this article online at http://dx.doi.org/10.1016/j.cub.2015.12.073.

AUTHOR CONTRIBUTIONS

Conceptualization, K.T.-K., S.B., and M.B.; Methodology, K.T.-K., S.B., M.B., and S.A.W.; Formal Analysis, S.B. and M.B.; Investigation, K.T.-K., S.B., M.B., and A.S.; Resources, J.G., P.E.C., M.S., and T.H.; Writing – Original Draft, K.T.-K.; Writing – Review & Editing, K.T.-K., S.B., and M.B.; Visualization, S.B.; Supervision, K.T.-K.; Project Administration, K.T.-K.; Funding Acquisition, K.T.-K.

ACKNOWLEDGMENTS

We thank Agnieszka T. Piszczeks and her team at IMBA for histological advice and expertise. We thank Kerstin Klien for taking care of our mice. We are grateful for the *(Tg)Stop/Rec8-Myc* mice provided by Nobuaki R. Kudo and *Rosa26-LacZ* mice provided by Elizabeth Robertson. We also thank Kim Nasmyth for fruitful discussions; the *Gdf9*-iCre experiments were initiated in his laboratory. This work was funded by the Austrian Academy of Sciences and by the European Research Council (ERC-StG-336460 ChromHeritance) to K.T.-K.

CellPress

Received: February 16, 2015
Revised: November 20, 2015
Accepted: December 31, 2015
Published: February 18, 2016

REFERENCES

1. Nasmyth, K., and Haering, C.H. (2009). Cohesin: its roles and mechanisms. Annu. Rev. Genet. *43*, 525–558.

2. Watanabe, Y., and Nurse, P. (1999). Cohesin Rec8 is required for reductional chromosome segregation at meiosis. Nature *400*, 461–464.

3. Tachibana-Konwalski, K., Godwin, J., van der Weyden, L., Champion, L., Kudo, N.R., Adams, D.J., and Nasmyth, K. (2010). Rec8-containing cohesin maintains bivalents without turnover during the growing phase of mouse oocytes. Genes Dev. *24*, 2505–2516.

4. Lee, J., Iwai, T., Yokota, T., and Yamashita, M. (2003). Temporally and spatially selective loss of Rec8 protein from meiotic chromosomes during mammalian meiosis. J. Cell Sci. *116*, 2781–2790.

5. Revenkova, E., Eijpe, M., Heyting, C., Gross, B., and Jessberger, R. (2001). Novel meiosis-specific isoform of mammalian SMC1. Mol. Cell. Biol. *21*, 6984–6998.

6. Revenkova, E., Eijpe, M., Heyting, C., Hodges, C.A., Hunt, P.A., Liebe, B., Scherthan, H., and Jessberger, R. (2004). Cohesin SMC1 beta is required for meiotic chromosome dynamics, sister chromatid cohesion and DNA recombination. Nat. Cell Biol. *6*, 555–562.

7. Ström, L., Karlsson, C., Lindroos, H.B., Wedahl, S., Katou, Y., Shirahige, K., and Sjögren, C. (2007). Postreplicative formation of cohesion is required for repair and induced by a single DNA break. Science *317*, 242–245.

8. Unal, E., Heidinger-Pauli, J.M., and Koshland, D. (2007). DNA double-strand breaks trigger genome-wide sister-chromatid cohesion through Eco1 (Ctf7). Science *317*, 245–248.

9. Kuleszewicz, K., Fu, X., and Kudo, N.R. (2013). Cohesin loading factor Nipbl localizes to chromosome axes during mammalian meiotic prophase. Cell Div. *8*, 12.

10. Visnes, T., Giordano, F., Kuznetsova, A., Suja, J.A., Lander, A.D., Calof, A.L., and Ström, L. (2014). Localisation of the SMC loading complex Nipbl/Mau2 during mammalian meiotic prophase I. Chromosoma *123*, 239–252.

11. Revenkova, E., Herrmann, K., Adelfalk, C., and Jessberger, R. (2010). Oocyte cohesin expression restricted to predictyate stages provides full fertility and prevents aneuploidy. Curr. Biol. *20*, 1529–1533.

12. Hodges, C.A., Revenkova, E., Jessberger, R., Hassold, T.J., and Hunt, P.A. (2005). SMC1beta-deficient female mice provide evidence that cohesins are a missing link in age-related nondisjunction. Nat. Genet. *37*, 1351–1355.

13. Hassold, T., and Chiu, D. (1985). Maternal age-specific rates of numerical chromosome abnormalities with special reference to trisomy. Hum. Genet. *70*, 11–17.

14. Hassold, T., and Hunt, P. (2001). To err (meiotically) is human: the genesis of human aneuploidy. Nat. Rev. Genet. *2*, 280–291.

15. Ottolini, C.S., Newnham, L.J., Capalbo, A., Natesan, S.A., Joshi, H.A., Cimadomo, D., Griffin, D.K., Sage, K., Summers, M.C., Thornhill, A.R., et al. (2015). Genome-wide maps of recombination and chromosome segregation in human oocytes and embryos show selection for maternal recombination rates. Nat. Genet. *47*, 727–735.

16. Kouznetsova, A., Lister, L., Nordenskjöld, M., Herbert, M., and Höög, C. (2007). Bi-orientation of achiasmatic chromosomes in meiosis I oocytes contributes to aneuploidy in mice. Nat. Genet. *39*, 966–968.

17. Nagaoka, S.I., Hassold, T.J., and Hunt, P.A. (2012). Human aneuploidy: mechanisms and new insights into an age-old problem. Nat. Rev. Genet. *13*, 493–504.

18. Tsutsumi, M., Fujiwara, R., Nishizawa, H., Ito, M., Kogo, H., Inagaki, H., Ohye, T., Kato, T., Fujii, T., and Kurahashi, H. (2014). Age-related decrease of meiotic cohesins in human oocytes. PLoS ONE *9*, e96710.

19. Sakakibara, Y., Hashimoto, S., Nakaoka, Y., Kouznetsova, A., Höög, C., and Kitajima, T.S. (2015). Bivalent separation into univalents precedes age-related meiosis I errors in oocytes. Nat. Commun. *6*, 7550, http://dx.doi.org/10.1038/ncomms8550.

20. Chiang, T., Duncan, F.E., Schindler, K., Schultz, R.M., and Lampson, M.A. (2010). Evidence that weakened centromere cohesion is a leading cause of age-related aneuploidy in oocytes. Curr. Biol. *20*, 1522–1528.

21. Lister, L.M., Kouznetsova, A., Hyslop, L.A., Kalleas, D., Pace, S.L., Barel, J.C., Nathan, A., Floros, V., Adelfalk, C., Watanabe, Y., et al. (2010). Age-related meiotic segregation errors in mammalian oocytes are preceded by depletion of cohesin and Sgo2. Curr. Biol. *20*, 1511–1521.

22. Uhlmann, F., Lottspeich, F., and Nasmyth, K. (1999). Sister-chromatid separation at anaphase onset is promoted by cleavage of the cohesin subunit Scc1. Nature *400*, 37–42.

23. Gerlich, D., Koch, B., Dupeux, F., Peters, J.M., and Ellenberg, J. (2006). Live-cell imaging reveals a stable cohesin-chromatin interaction after but not before DNA replication. Curr. Biol. *16*, 1571–1578.

24. Ivanov, D., and Nasmyth, K. (2007). A physical assay for sister chromatid cohesion in vitro. Mol. Cell *27*, 300–310.

25. Hayashi, S., Lewis, P., Pevny, L., and McMahon, A.P. (2002). Efficient gene modulation in mouse epiblast using a Sox2Cre transgenic mouse strain. Mech. Dev. *119* (*Suppl 1*), S97–S101.

26. Lan, Z.J., Xu, X., and Cooney, A.J. (2004). Differential oocyte-specific expression of Cre recombinase activity in GDF-9-iCre, Zp3cre, and Msx2Cre transgenic mice. Biol. Reprod. *71*, 1469–1474.

27. Reddy, P., Liu, L., Adhikari, D., Jagarlamudi, K., Rajareddy, S., Shen, Y., Du, C., Tang, W., Hämäläinen, T., Peng, S.L., et al. (2008). Oocyte-specific deletion of Pten causes premature activation of the primordial follicle pool. Science *319*, 611–613.

28. Adhikari, D., Zheng, W., Shen, Y., Gorre, N., Hämäläinen, T., Cooney, A.J., Huhtaniemi, I., Lan, Z.J., and Liu, K. (2010). Tsc/mTORC1 signaling in oocytes governs the quiescence and activation of primordial follicles. Hum. Mol. Genet. *19*, 397–410.

29. Andreu-Vieyra, C.V., Chen, R., Agno, J.E., Glaser, S., Anastassiadis, K., Stewart, A.F., and Matzuk, M.M. (2010). MLL2 is required in oocytes for bulk histone 3 lysine 4 trimethylation and transcriptional silencing. PLoS Biol. *8*, e1000453, http://dx.doi.org/10.1371/journal.pbio.1000453.

30. Sen, A., and Hammes, S.R. (2010). Granulosa cell-specific androgen receptors are critical regulators of ovarian development and function. Mol. Endocrinol. *24*, 1393–1403.

31. Li, X., Tripurani, S.K., James, R., and Pangas, S.A. (2012). Minimal fertility defects in mice deficient in oocyte-expressed Smad4. Biol. Reprod. *86*, 1–6.

32. Yu, C., Zhang, Y.L., Pan, W.W., Li, X.M., Wang, Z.W., Ge, Z.J., Zhou, J.J., Cang, Y., Tong, C., Sun, Q.Y., and Fan, H.Y. (2013). CRL4 complex regulates mammalian oocyte survival and reprogramming by activation of TET proteins. Science *342*, 1518–1521.

33. Jiang, Z.Z., Hu, M.W., Wang, Z.B., Huang, L., Lin, F., Qi, S.T., Ouyang, Y.C., Fan, H.Y., Schatten, H., Mak, T.W., and Sun, Q.Y. (2014). Survivin is essential for fertile egg production and female fertility in mice. Cell Death Dis. *5*, e1154, http://dx.doi.org/10.1038/cddis.2014.126.

34. Nashun, B., Hill, P.W., Smallwood, S.A., Dharmalingam, G., Amouroux, R., Clark, S.J., Sharma, V., Ndjetehe, E., Pelczar, P., Festenstein, R.J., et al. (2015). Continuous histone replacement by Hira is essential for normal transcriptional regulation and de novo DNA methylation during mouse oogenesis. Mol. Cell *60*, 611–625, http://dx.doi.org/10.1016/j.molcel.2015.10.010.

35. Soriano, P. (1999). Generalized lacZ expression with the ROSA26 Cre reporter strain. Nat. Genet. *21*, 70–71.

36. Keeney, S., Giroux, C.N., and Kleckner, N. (1997). Meiosis-specific DNA double-strand breaks are catalyzed by Spo11, a member of a widely conserved protein family. Cell *88*, 375–384.

37. Hirota, T., Ohta, H., Shigeta, M., Niwa, H., and Saitou, M. (2011). Drug-inducible gene recombination by the Dppa3-MER Cre MER transgene in the developmental cycle of the germ cell lineage in mice. Biol. Reprod. *85*, 367–377.

38. Zhang, Y., Riesterer, C., Ayrall, A.M., Sablitzky, F., Littlewood, T.D., and Reth, M. (1996). Inducible site-directed recombination in mouse embryonic stem cells. Nucleic Acids Res. *24*, 543–548.

39. Lee, J., Yokota, T., and Yamashita, M. (2002). Analyses of mRNA expression patterns of cohesin subunits Rad21 and Rec8 in mice: germ cell-specific expression of rec8 mRNA in both male and female mice. Zoolog. Sci. *19*, 539–544.

40. Heidinger-Pauli, J.M., Mert, O., Davenport, C., Guacci, V., and Koshland, D. (2010). Systematic reduction of cohesin differentially affects chromosome segregation, condensation, and DNA repair. Curr. Biol. *20*, 957–963.

Current Biology, Volume *26*

Supplemental Information

Chromosome Cohesion Established by Rec8-Cohesin in Fetal Oocytes Is Maintained without Detectable Turnover in Oocytes Arrested for Months in Mice

Sabrina Burkhardt, Máté Borsos, Anna Szydlowska, Jonathan Godwin, Suzannah A. Williams, Paula E. Cohen, Takayuki Hirota, Mitinori Saitou, and Kikuë Tachibana-Konwalski

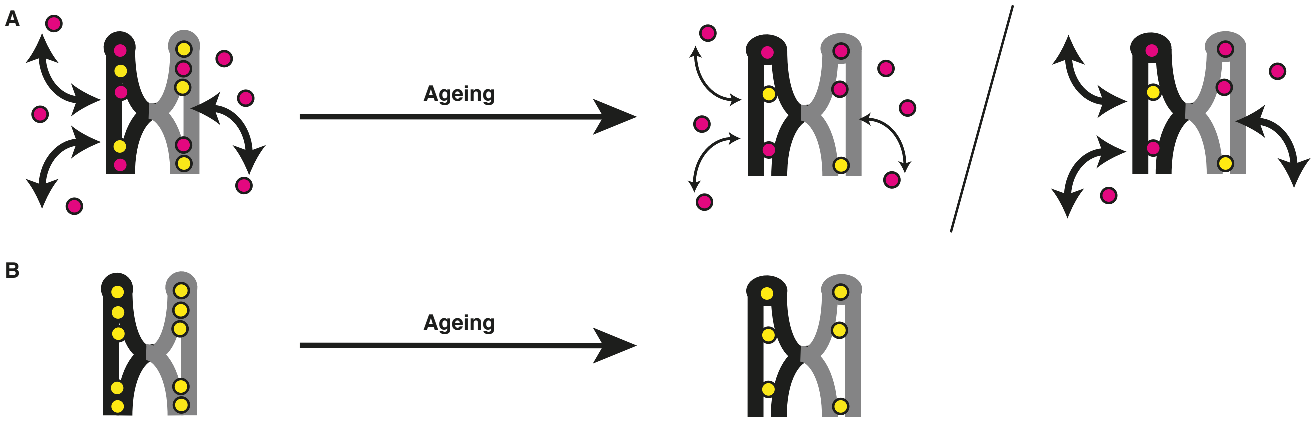

Figure S1

Figure S1. Related to Figure 1.

Model of cohesion maintenance in aging oocytes.

Models of cohesion maintenance with or without turnover to explain the decrease in chromosomal cohesin and increase in chromosome segregation errors with age. Maternal and paternal homologous chromosomes (black and grey) are held together by chiasmata mediated by cohesin distal to crossover sites.

(A) Cohesion is established during meiotic S phase (yellow) and maintained with turnover as new cohesive structures are built after S phase (red). The cohesion establishment mechanism becomes defective (black arrows) or cohesin complexes needed for replenishment (red) deteriorate in ageing oocytes.

(B) Cohesion is established during meiotic S phase (yellow) and maintained without turnover. Chromosomal cohesin decays and is irreplaceably lost from chromosomes in ageing oocytes.

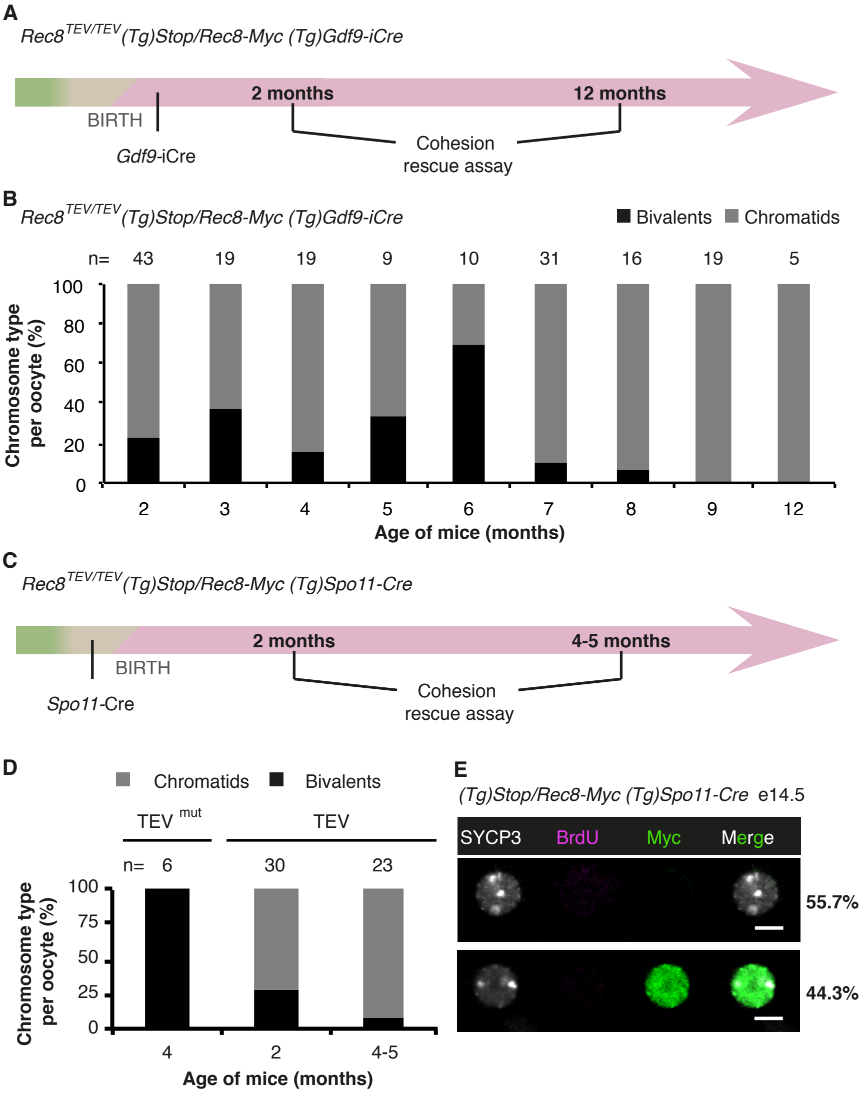
A
Rec8 TEV/TEV (Tg)Stop/Rec8-Myc (Tg)Gdf9-iCre
BIRTH
2 months
12 months
Gdf9-iCre
Cohesion rescue assay
B
Rec8 TEV/TEV (Tg)Stop/Rec8-Myc (Tg)Gdf9-iCre
Bivalents
Chromatids
n= 43 19 19 9 10 31 16 19 5
Chromosome type per oocyte (%)
100 80 60 40 20 0
2 3 4 5 6 7 8 9 12
Age of mice (months)
C
Rec8 TEV/TEV (Tg)Stop/Rec8-Myc (Tg)Spo11-Cre
BIRTH
2 months
4-5 months
Spo11-Cre
Cohesion rescue assay
D
Chromatids
Bivalents
TEV mut
TEV
n= 6 30 23
Chromosome type per oocyte (%)
100 75 50 25 0
4 2 4-5
Age of mice (months)
E
(Tg)Stop/Rec8-Myc (Tg)Spo11-Cre e14.5
SYCP3
BrdU
Myc
Merge
55.7%
44.3%

Figure S2

Figure S2. Related to Figure 2; Table S1.

***De novo* expression of Rec8-Myc using *Gdf9*-iCre or *Spo11*-Cre results in generation of cohesive structures during meiotic S phase and homologous recombination.**

(A) Timing of cohesion rescue assay utilizing *Gdf9*-iCre to activate Rec8-Myc in oocytes shortly after birth. Green, meiotic DNA replication; beige, homologous recombination; pink, dictyate stage. See also Figure 2 and Table S1 for evaluation of accurate deletion timing of *Gdf9*-iCre.

(B) Quantification of cohesion rescue assay performed with *Rec8*$^{TEV/TEV}$ *(Tg)Stop/Rec8-Myc (Tg)Gdf9-iCre* oocytes from females ranging from 2-12 months. The cell number for each time point is indicated (n). Oocytes were obtained from > 1 female for all time points except 8 and 12 months.

(C) Since the Spo11 endonuclease produces the DSBs that initiate homologous recombination, we considered using a Cre recombinase under the *Spo11* promoter to activate *Rec8-Myc*. Green, meiotic DNA replication; beige, homologous recombination; pink, dictyate stage. See also Figure 2 and Table S1 for evaluation of accurate deletion timing of *Spo11*-Cre.

(D) Activation of Rec8-Myc by *Spo11*-Cre results in generation of cohesive structures. Quantification of cohesion rescue assay in *Rec8*$^{TEV/TEV}$ *(Tg)Stop/Rec8-Myc (Tg)Spo11-Cre* oocytes. Oocytes were obtained from > 3 females for all time points using TEV protease.

(E) *Spo11*-Cre is active during meiotic recombination. e14.5 *(Tg)Stop/Rec8-Myc (Tg)Spo11-Cre* embryonic ovaries were scored for chromosomal Rec8-Myc staining in recombining (SYCP3-positive) germ cells, mean is given, SEM=1.6%, n>1000 cells from 7 females. Scale bar, 10 μm.

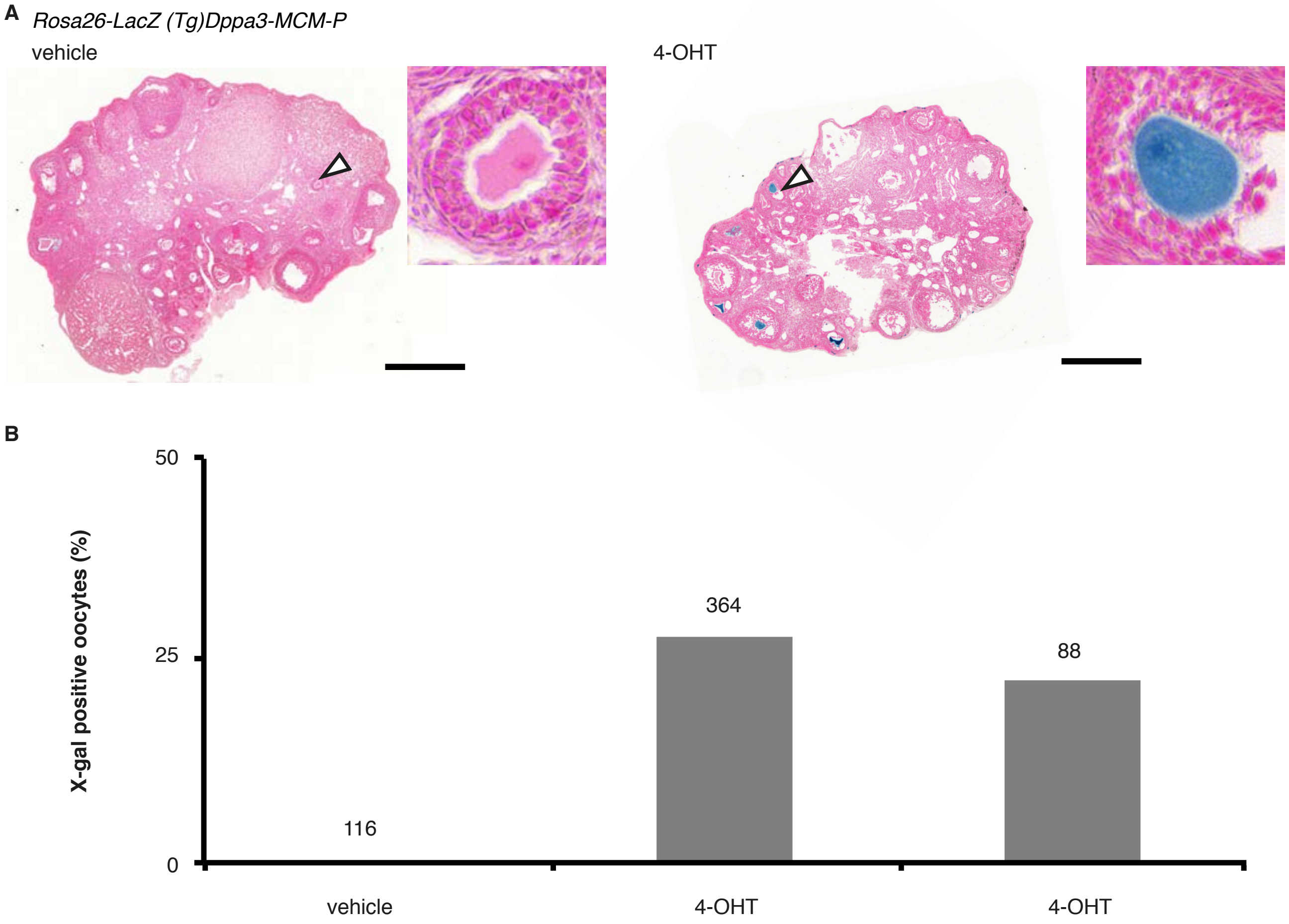
A
Rosa26-LacZ (Tg)Dppa3-MCM-P
vehicle
4-OHT
B
X-gal positive oocytes (%)
50
25
0
116
364
88
vehicle
4-OHT
4-OHT

Figure S3

Figure S3. Related to Figure 3; Figure 4; Table S1.

Oocyte-specific deletion efficiency of *(Tg)Dppa3*-MCM-P in adult ovary.

(A) Representative histological sections of individual *Rosa26-LacZ (Tg)Dppa3-MCM-P* mice after treatment with vehicle or 4-OHT. Arrowhead, magnified oocyte. Scale bar, 500 µm.

(B) Oocytes larger than 30 µm were scored. Total cell numbers are indicated.

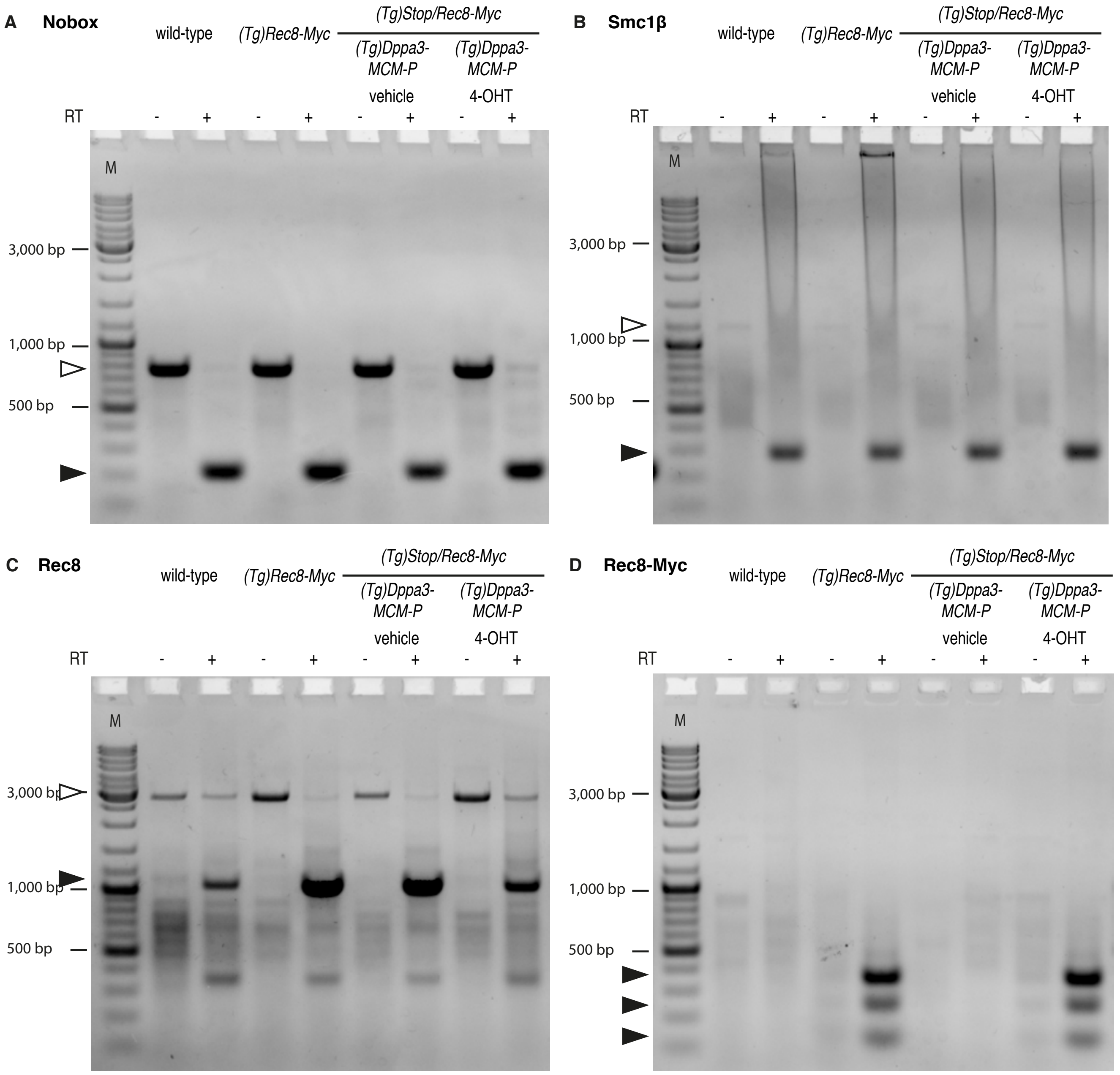
A Nobox
wild-type
(Tg)Rec8-Myc
(Tg)Stop/Rec8-Myc
(Tg)Dppa3-MCM-P
vehicle
(Tg)Dppa3-MCM-P
4-OHT
RT
- + - + - + - +
M
3,000 bp
1,000 bp
500 bp
B Smc1β
wild-type
(Tg)Rec8-Myc
(Tg)Stop/Rec8-Myc
(Tg)Dppa3-MCM-P
vehicle
(Tg)Dppa3-MCM-P
4-OHT
RT
- + - + - + - +
M
3,000 bp
1,000 bp
500 bp
C Rec8
wild-type
(Tg)Rec8-Myc
(Tg)Stop/Rec8-Myc
(Tg)Dppa3-MCM-P
vehicle
(Tg)Dppa3-MCM-P
4-OHT
RT
- + - + - + - +
M
3,000 bp
1,000 bp
500 bp
D Rec8-Myc
wild-type
(Tg)Rec8-Myc
(Tg)Stop/Rec8-Myc
(Tg)Dppa3-MCM-P
vehicle
(Tg)Dppa3-MCM-P
4-OHT
RT
- + - + - + - +
M
3,000 bp
1,000 bp
500 bp

Figure S4

Figure S4. Related to Figure 4.

Detection of Rec8-Myc transcript in adult ovary.

(A-D) Rec8-Myc mRNA is detectable in ovary of 4-OHT treated *(Tg)Stop/Rec8-Myc (Tg)Dppa3-MCM-P* mice. Entire agarose gels for mRNA detection of (A) Nobox, (B) Smc1β, (C) Rec8, and (D) Rec8-Myc by RT-PCR from ovary of wild-type, *(Tg)Rec8-Myc*, and *(Tg)Stop/Rec8-Myc (Tg)Dppa3-MCM-P* (vehicle or 4-OHT treated) mice are displayed. Black arrowhead, amplicon size for mRNA detection. White arrowhead, amplicon size for genomic DNA. RT, reverse transcriptase. M, DNA ladder.

Table S1. Related to Figure 2; Figure 3; Figure 4; Figure S2; Figure S4.

Overview of the deletion efficiency of female germline specific *(Tg)Cre* lines evaluated by up to 3 different approaches.

	**Deletion timing**		**Deletion efficiency**		
	**Developmental timing**	**Cell cycle stage**	**X-gal positive oocytes[a]**	**Genetic analysis[b]**	**Single cell PCR[c]**
(Tg)Gdf9-iCre [S1]	e13.5 *	Meiotic S phase, recombination	-	90% (20 pups)	-
(Tg)Spo11-Cre	e13.5	Meiotic S phase, recombination	94% (2♀)	100% (18 pups)	89% (53 cells/10♀)
(Tg)Dppa3-MCM-P [S2]	Inducible	Inducible	25% (2♀)	28% (46 pups)	46% (71 cells/8♀)

* High inter-cell & inter-mouse variability

[a] X-gal positive oocytes were scored in relation to unstained oocytes in adult ovary obtained from *Rosa26-LacZ* crosses to the respective *(Tg)Cre* line. Negative controls (*Rosa26-LacZ*, or vehicle treated *Rosa26-LacZ (Tg)Dppa3-MCM-P*) showed no X-gal positive staining.

[b] Deletion efficiency was determined by genotyping F1 offspring of *(Tg)Stop/Rec8-Myc (Tg)Cre* x *wild-type* crosses for deleted *Stop* cassette.

[c] Single-cell genotyping for deletion of *Stop* cassette was performed in context of live cell imaging experiments using single oocytes from *Rec8*$^{TEV/TEV}$*(Tg)Stop/Rec8-Myc (Tg)Cre* mice.

Supplemental Experimental Procedures

Mice, 4-hydroxy-tamoxifen injections, Caesarean section, genotyping

The care and use of the mice were in accordance with the guidelines of the International guiding principles for biomedical research involving animals (CIOMS, the Council for International Organizations of Medical Sciences). All of the studies were performed on a mixed BL6/129SV genetic background. Cre expression in *(Tg)Dppa3-MCM-P* mice was induced by two consecutive intraperitoneal injections within four days of 3 mg 4-OHT (Sigma, H6278) dissolved in sunflower oil (Sigma, S5007) as originally described [S2]. *Dppa3*-MCM-P expression in e10.5 embryos was induced by a single intraperitoneal injection of pregnant females with 2 mg 4-OHT. Caesarean sections on pregnant females were performed on e19.0. Once the pregnant females were killed by cervical dislocation, the abdomen was opened and each uterine horn was opened carefully with forceps. The pups and placenta were gently extruded and the amniotic sac opened. Amniotic fluid was removed by softly rubbing with a paper towel until the pups breathed. After disconnecting the umbilical cord the pups were placed with foster mothers. Post-experimental genotyping for deletion of STOP cassette in single oocytes was performed using KAPA Mouse Genotyping kit (KK7352).

In vitro culture, microinjection, and time-lapse confocal microscopy

Fully grown mouse GV oocytes were isolated, cultured, and injected as described previous [S3]. Briefly, injection of mRNA for H2B-mCherry and CenpB-EGFP was performed to monitor chromosomes and centromeres, respectively. Injection of TEV^{mut} or TEV was carried out for cohesin turnover analysis. IBMX was washed out 2 hours after injection to resume meiosis. Chromosome type in oocytes was scored as containing either 20 bivalent chromosomes or at least 72 single chromatids (and no bivalents) by 5 h post-GVBD. Imaging was performed as described previously [S3]. Briefly, a customized Zeiss LSM510 META confocal microscope equipped with P C-Apochromat 63x/1.2 NA water immersion objective lens was used for image acquisition. Chromosomes labeled with H2B-mCherry were tracked with an EMBL-developed tracking macro adapted to our microscope [S4]. Image stacks of 11 slices of 2 µm were captured every 30 min.

X-Gal staining of adult and embryonic ovaries, histology

Fixation was performed for 5 hours at 4 °C for adult, and for 20 minutes at RT for embryonic gonads in 0.2% glutaraldehyde, 2 mM $MgCl_2$ in PBS. Gonads were washed three times in wash solution (2 mM MgCl2, 0.1% sodium deoxycholate, 0.2% nonidet P-40 in 0.1 M NaPi (pH 7.4) buffer) and stained in wash solution containing (5 mM potassium ferrocyanide, 5mM potassium ferricyanide, 1 mg/ml of X-gal (Sigma, B4252) overnight at 37 °C. Next day, gonads were washed 3 times in wash solution and left in 70% ethanol until conventional histology processing. 3 µm thick sections were counterstained with Nuclear Fast Red and imaged with a Mirax Slide Scanner (Zeiss) and analyzed in Panoramic Viewer (3DHistotech).

Meiotic prophase spreading and *in situ* staining

To obtain meiotic prophase oocytes, embryonic ovaries of different aged embryos (e13.5-17.5) were isolated. For BrdU pulse labeling, pregnant females were injected IP with 50 mg/kg BrdU (Invitrogen) dissolved in embryo water (Sigma, W1503) 30 min before embryo isolation. Spreads were prepared using methods previously described [S5] with one exception: when preparing cells for DDX4 staining, in order to preserve the cytoplasmic protein fraction 2% PFA solution without 0.2% Triton-X 100 was used. Immunofluorescent staining was performed using rabbit anti-SYCP3 (Abcam, ab15093), rabbit anti-DDX4 (Abcam, ab13840), rat anti-BrdU (Abcam, ab6326), mouse anti-Myc-tag (for detection of Rec8-Myc) (Millipore, 05-724) primary antibodies, appropriate Alexa488/568/647 conjugated secondary antibodies (Invitrogen) were used for visualization, 1 µg/ml DAPI was applied for DNA counterstaining.

Imaging of spreads was performed on a Zeiss LSM780 confocal microscope equipped with 63x/1.4 plan-apochromat Oil DIC or 40x/1.4 EC plan-apochromat Oil DIC objective lenses.

Metaphase I chromosome spreads

Oocytes were washed through Tyrode's buffer (pH 2.5) at 37 °C to remove zona pellucidae. After subsequent 18 min incubation in 1:1 FBS:water at 37 °C, oocytes were fixed in a drop of 1% paraformaldehyde with 0.15% Triton X-100 and 3 mM DTT on a glass slide. After air-drying, oocytes were incubated with primary antibodies overnight at 4 °C, and then appropriate Alexa488/568/647 conjugated secondary antibodies (Invitrogen) were used for visualization, 1 µg/ml DAPI was applied for DNA counterstaining. Samples were examined on a Zeiss LSM780 confocal microscope equipped with 63x/1.4 plan-apochromat Oil DIC objective lenses. 3D surface plots of pixel intensities were generated with ImageJ.

RT-PCR

For RNA preparation, adult ovaries or testes were snapfrozen in liquid nitrogen and were grinded using glass dounce homogenizers. RNA extraction was performed with RNeasy Mini Kit (Qiagen) according to the manufactures instruction. RT reaction was carried out with SuperscriptIII (Life Technologies) using 18mer oligo dT primers (Thermo Scientific). Transcripts were detected by PCR: Rec8 (*q.v.* [S6, S7]), Nobox and Smc1β (*q.v.* [S8]), Rec8-Myc (ATCTGCTCTTGGTGCTGTCC, CAGAAATCAACTTTTGTTCACCAC).

Supplemental References

S1 Lan, Z.J., Xu, X., Cooney, A.J. (2004). Differential oocyte-specific expression of Cre recombinase activity in GDF-9-iCre, Zp3cre, and Msx2Cre transgenic mice. Biol. Reprod. *71*, 1469-1474.

S2 Hirota, T., Ohta, H., Shigeta, M., Niwa, H., Saitou, M. (2011). Drug-inducible gene recombination by the Dppa3-MER Cre MER transgene in the developmental cycle of the germ cell lineage in mice. Biol. Reprod. *85*, 367-377.

S3 Tachibana-Konwalski, K., Godwin, J., van der Weyden, L., Champion, L., Kudo, N.R., Adams, D.J., Nasmyth, K. (2010). Rec8-containing cohesin maintains bivalents without turnover during the growing phase of mouse oocytes. Genes. Dev. *24*, 2505-2516.

S4 Rabut, G., and Ellenberg, J. (2004). Automatic real-time three-dimensional cell tracking by fluorescence microscopy. J. Microsc. *216*, 131-137.

S5 Susiarjo, M., Rubio, C., Hunt, P. (2009). Analysing mammalian female meiosis. Methods Mol. Biol. *558*, 339-354.

S6 Lee, J., Yokota, T., Yamashita, M. (2002). Analyses of mRNA expression patterns of cohesin subunits Rad21 and Rec8 in mice: germ cell-specific expression of rec8 mRNA in both male and female mice. Zoolog. Sci. *19*, 539-544.

S7 Bannister, L.A., Reinholdt, L.G., Munroe, R.J., Schimenti, J.C. (2004). Positional cloning and characterization of mouse mei8, a disrupted allelle of the meiotic cohesin Rec8. Genesis *40*, 184-194.

S8 Chiang, T., Duncan, F.E., Schindler, K., Schultz, R.M., Lampson, M.A. (2010). Evidence that weakened centromere cohesion is a leading cause of age-related aneuploidy in oocytes. Curr. Biol. *20*, 1522-1528.
